# Origin and variability of statistical dependencies between peak, volume, and duration of rainfall-driven flood events

**DOI:** 10.1038/s41598-021-84664-1

**Published:** 2021-03-04

**Authors:** L. Rahimi, C. Deidda, C. De Michele

**Affiliations:** Department of Civil and Environmental Engineering, Politecnico di Milano, Milan, Italy

**Keywords:** Hydrology, Natural hazards

## Abstract

Floods are among the most common and impactful natural events. The hazard of a flood event depends on its peak (Q), volume (V) and duration (D), which are interconnected to each other. Here, we used a worldwide dataset of daily discharge, two statistics (Kendall’s tau and Spearman’s rho) and a conceptual hydrological rainfall-runoff model as model-dependent realism, to investigate the factors controlling and the origin of the dependence between each couple of flood characteristics, with the focus to rainfall-driven events. From the statistical analysis of worldwide dataset, we found that the catchment area is ineffective in controlling the dependence between Q and V, while the dependencies between Q and D, and V and D show an increasing behavior with the catchment area. From the modeling activity, on the U.S. subdataset, we obtained that the conceptual hydrological model is able to represent the observed dependencies between each couple of variables for rainfall-driven flood events, and for such events, the pairwise dependence of each couple is not causal, is of spurious kind, coming from the “Principle of Common Cause”.

## Introduction

Characterizing the response of a catchment to extreme meteorological events is of considerable importance for evaluating the flood risk^1,2^, designing flood protection structures^3^, and assessing the reliability of existing hydraulic engineering works^4^. The capability of predicting the response of a catchment to extreme meteorological events and/or identifying the similarity of response between catchments is an important issue for flood studies. In the regional context, the similarity of catchment response to precipitation input can allow transfering the information from gauged to ungauged catchments. For this, the concept of “catchment similarity” has gained increasing interest in the last two decades^5–9^, even if the first studies are dated back to the end of 80s^10,11^. The flood event can be characterized through some main variables like flood peak (Q), volume (V), and duration (D), which are dependent each other^4,12–15^. Karmakar and Simonovic^15^ argued that these variables are mutually dependent, because they belong to the same physical phenomenon. Some studies have investigated the dependence between these couples, some using model-based approaches^16,17^, others using data-based approaches^18–20^. The importance of such dependencies is fundamental to properly describe the magnitude of a flood, because neglecting these dependencies can lead to under- or over-estimation of flood magnitude^13,21^. Other studies have used the pairwise dependence of peak, volume and duration to build bivariate^4,13–15,22–24^ and multivariate probability models^16,25–27^, mainly through the use of copula concept^28^. Vezzoli et al.^29^ used the pairwise dependence in order to assess the performance of climate-hydrology models. Some studies have investigated the possible non-stationarity of such dependencies^30–32^.

Specifically, Karmakar and Simonovic^15^ analyzed the pairwise dependence between peak, volume, and duration, of flood events identified using different discharge thresholds, using the streamflow data of Red River at Grand Forks, North Dakota (USA). The pairwise dependence was described through the Pearson's linear correlation coefficient, as well as, non-parametric rank-based measures of dependence like the Kendall’s tau and Spearman’s rho, as suggested by^28^. Karmakar and Simonovic^15^ found that (1) the dependence of Q–D and V–D couples was very sensitive, and increasing with increasing the discharge threshold; (2) the Q–V dependence is high for all the discharge thresholds; (3) the Q–V dependence was greater than the one observed for Q–D and V–D couples, fact also observed by^16,25^.

Serinaldi and Kilsby^18^ have investigated the nature of the dependence between each pair of flood event variables identified using different discharge thresholds (80th, 90th, and 95th percentile) considering 80 daily time series of discharge recorded in the continental United States, and bootstrap algorithms. They found that the pairwise dependence can be reproduced using a univariate bootstrapping of discharge without recurring to multivariate techniques. They concluded that the pairwise dependence can be explained as the result of summing independent random variables over random durations.

Gaál et al.^19^ have investigated the factors influencing the degree of dependence between flood peak and volume, if it is possible to relate flood types and the Q–V dependence, considering 330 catchments in Austria, having an area ranging from 6 to 500 km^2^. They made a process-based analysis, using a classification of the rainfall-runoff events in three different typologies: synoptic, flash and snowmelt floods, following^33^. They considered the maximum annual flood discharges and the associated volumes, and the Spearman’s rho as measure of dependence. They found that climate-related factors (flood process types such as flash floods and snowmelt floods) are more important than catchment-related factors (elevation, catchment area) in controlling the Q–V dependence. In particular, they obtained Spearman’s coefficients in the range 0.2–0.8, with a weaker Q–V dependence in high alpine catchments due to the mix of flood types including long-duration snowmelt events, synoptic floods and flash floods, respect to lowlands where the flood durations vary less.

Szolgay et al.^20^ made a process-based analysis, distinguishing the different flood types, and studying the structure of Q–V dependence. They used the empirical copulas, and a goodness of fit test between empirical copulas. They addressed the following two questions: (1) How similar is the Q–V structure of dependence for different flood types for a given catchment? (2) How similar is the Q–V structure of dependence between catchments for a given flood type? They considered 72 catchments in the North-West of Austria, and globally 25,697 events categorized in synoptic, flash and snowmelt floods. They found that, for a given catchment, the empirical copula of flash flood events is more distinguishable from the empirical copula of synoptic and snowmelt events than the empirical copulas between these last. In addition, they found that there is an added value in dealing with separately flood types and in pooling events of the same type in a region when analysing the copula of peak-volume variables.

Grimaldi et al.^17^ addressed the concept of catchment compatibility, which is a little bit less general than the catchment similarity. In fact, the catchment compatibility relies on the possibility of transfering, from one catchment to another, the information on the pairwise dependence between variables like peak, volume and duration. They investigated via a hydrological (rainfall-runoff) model the variability of the pairwise dependence between peak, volume and duration with respect to the following catchment features: the concentration time Tc and the curve number CN, using the same climatic forcing (synthetic rainfall time series) and topography. They summarized the results in compatibility maps, which allow to understand which Tc-CN combinations are to be considered compatible in terms of pairwise dependence of flood event variables.

Thus, except for^18^, no studies have investigated the origin of the pairwise statistical dependence between the flood event variables (if there are causal statements between the variables), as well as, no worldwide assessments, but only catchment-based or country-based analyses, of such statistical dependencies are available in literature, to our knowledge. Here we want to address the following research questions: (1) What is the variability spectrum of the dependencies between the flood event variables? (2) What is the origin of such dependencies? Are there causal statements between the flood event variables, or the dependencies are of spurious type? In order to give answers to these questions, we decide to move in three different directions, using both data-based and model-based analyses: (1) calculate the pairwise statistical dependence of peak, volume, and duration of flood events using a worldwide database of daily discharge; (2) investigate if a crude conceptual rainfall-runoff model is able to reproduce the observed pairwise dependencies for rainfall-driven flood events, using the U.S. subdataset; (3) use the conceptual rainfall-runoff model as model-dependent realism in order to investigate the origin of pairwise dependencies for rainfall-driven flood events.

## Results

We considered the Global Runoff Database (GRDC) of daily river discharge, including more than 4638 stations spanned in eighty countries, over the period 1812–2014. The time series of discharge are not contemporaneous, however we have tested the series against the presence of trends, or changing points, as mentioned in the next. The stations are characterized by a contributing area ranging between 0.07 km^2^ and 4,680,000 km^2^, however, since the temporal resolution is one day, we have not considered in the analysis small catchments with temporal dynamics within the day, roughly saying catchments with an area smaller than 500 km^2^. For each station, the flood events and flood characteristics, namely peak Q, volume V and duration D, have been selected using a threshold-based approach, and a criterion of temporal independence of flood events (see Materials and Methods section and Fig. S1). We have considered: 80th, 90th, and 95th percentile of daily discharge as threshold values. We selected these because we intend to consider the rainfall-driven component of flood events. Increasing the threshold value, we progressively leave out the processes which produce the base flow (i.e., snow and ice melting, subsurface or groundwater flow), considering only the catchment response to rainfall forcing.

The extracted time series of Q, V and D, have ties, inevitably present having used a daily time resolution and 1 m^3^/s resolution for the discharge. We found more ties in the time series of D, respect to the time series of Q and V. In addition, increasing the threshold value, increases the probability of occurrence of a flood event with smaller duration and thus number of "ties" in the time series of D. These ties could impact in the calculation of pairwise dependencies, and in particular V–D, and Q–D dependencies. Following^34^, we have used a randomization technique, also known as “jittering”, to remove repeated values and provide the natural granularity of the observed phenomenon. In particular, we used a Uniform distribution to randomize the ties, because it is an “noninformative” distribution, and in order to avoid unnecessary assumptions associated to this randomization.

The extracted data, for each specific threshold level, was checked against the presence of trends, or changing points, respectively through Mann–Kendall test, and Pettitt test, as well as, the stochastic independence of events in each station through the autocorrelation function, with a 1% significance level (details in Materials and Methods Section). Then, we focused the attention to the stations with at least 40 data, for each threshold, in order to have enough data to estimate the measures of dependence. Figs. S2–S4 provide the location of the stations considered, with and without at least 40 events, for the different thresholds, respectively 80th, 90th, 95th percentile.

Table 1 shows, for each threshold, the number of stations that passed all statistical tests, and those with at least 40 data: from 1310 to 1922 (i.e., from about 28% to 41% of the dataset) increasing the threshold from 80 to 95th percentile, and similarly the number of stations with at least 40 data, passed from 1140 to 1411 (i.e., from 25 to 30% of dataset). This depends on the threshold, and the fact that, at high thresholds the probability of test rejection and the dependence between events are less common than at low thresholds^35^.

**Table 1:** The number of stations at different thresholds, and with at least 40 flood events.

Threshold (percentile)	80th	90th	95th
N. of stations	1310 (28%)	1602 (35%)	1922 (41%)
N. of stations (with at least 40 events)	1140 (25%)	1316 (28%)	1411 (30%)

Then, we calculated the Spearman’s rho, and the Kendall’s tau for each couple of variables (Q, V), (V, D) and (Q, D), in each station, and for each threshold (see Supplementary material, Table S1). We have investigated the impact of the criterion of temporal independence of flood events on the estimation of the pairwise dependence (both in terms of Kendall’s tau and Spearman’s rho). In particular, for 80th, 90th, and 95th percentile, respectively in Figs. S5,S6,S7, we have compared the pairwise dependence considering and not considering such criterion. From Figs. S5–S7, it is possible to see that the criterion of temporal independence of flood events (considered in the next) does not affect significantly the estimation of pairwise dependence of Q, V and D.

From Table S1, the median value of the calculated statistics is quite high (say ${ \gtrsim }$ 0.6). For (Q, V), the median value of the Spearman’s rho is between 0.967 and 0.969, while the Kendall’s tau is between 0.855 and 0.856, for the considered thresholds. Similarly, for (V, D), the median value of the Spearman’s rho is between 0.902 and 0.941, and the Kendall’s tau is between 0.729 and 0.795. For (Q, D) the median value of the Spearman’s rho is between 0.795 and 0.855, while the Kendall’s tau is between 0.595 and 0.666. As a general comment, we found that the Q–V dependence is greater than Q–D and V–D dependence. This result is in agreement with those found by^15,16,25^. In addition, the estimate of Spearman’s rho resulted greater than the correspondent estimate of Kendall’s tau, for the different pairs and different thresholds.

**Figure 1:**
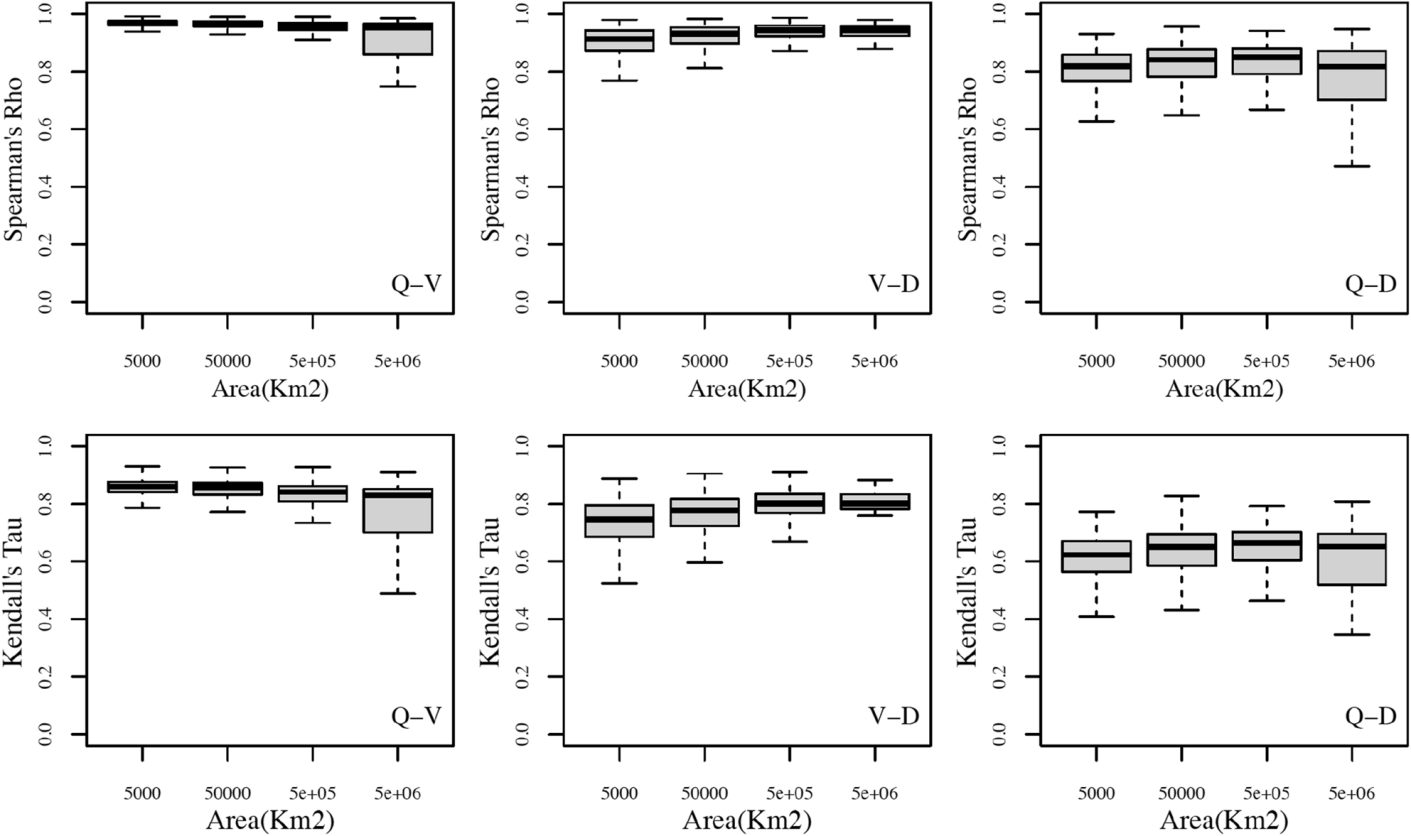
Boxplots of the measures of dependence (Spearman’s rho in upper panels and Kendall’s tau in lower panels) as function of the catchment area, for each couple of flood variables (Q–V in the left column, Q–D in the right column, V–D in the intermediate column), using stations with at least 40 events and the 90th percentile of daily discharge as threshold. The catchment area is divided in four classes: small (500, 5000] km^2^, medium (5000, 50,000] km^2^, large (50,000, 500,000] km^2^, and very large (500,000, 5,000,000] km^2^.

Figure 1 shows the box-plots of the Spearman’s rho (upper panels) and Kendall’s tau (lower panels) for the couples (Q, V) in the left panels, (V, D) in the central panels and (Q, D) in the right panels, as a function of catchment area, using the 90th percentile as threshold. In Fig. 1, the catchment area has been divided in four classes: small (500, 5000] km^2^, medium (5000, 50,000] km^2^, large (50,000, 500,000] km^2^, and very large (500,000, 5,000,000] km^2^. Figs. S8–S9, as supplement, show the boxplots of pairwise dependencies (respectively Spearman’s rho and Kendall’s tau) against the catchment area, for all the thresholds considered. From Fig. 1, as general comment, it is possible to say that the dependence between Q and V is practically constant with the catchment area, even if the boxplot correspondent to the last range of areas is wider due to its smaller sample. It seems surprisingly that the catchment area does not influence the dependence between Q and V. This result was also found by^19^. From Fig. 1, it is possible to see that the dependence between Q and D and V and D is slightly increasing with catchment area, except for the boxplot correspondent to the last range of areas for Q–D dependence (both for Spearman’s rho and Kendall’s tau cases), probably due to its smaller sample.

We have also considered the impact of the threshold, chosen for the identification of flood events, on the estimation of the pairwise dependence, both in terms of Kendall’s tau and Spearman’s rho. In particular, we compared the Kendall’s taus using 80th and 90th percentiles in Fig. S10, 80th and 95th percentiles in Fig. S11, and 90th and 95th percentiles in Fig. S12. From Figs. S10–S12, it is possible to see that the points are scattered around the 1:1 line, the highest differences are observed, when we pass from 80 to 95th percentiles (as expected) and in terms of the dependencies involving D variable (i.e., Q–D and V–D). Less influenced is the dependence between Q and V variables. Similar comment applies to Figs. S13–S15 for the Spearman’s rho.

Then, we considered a crude conceptual rainfall-runoff model, and checked if it is able to reproduce the observed pairwise dependencies of flood event variables, in the case of rainfall-driven flood events. For this model-based analysis, we restricted the attention to the continental United States, and in particular to 53 catchments (with a catchment area in the range 601–74,165 km^2^), where we have information about the precipitation dynamics. The catchment response is described through a linear reservoir. The storage constant k of the catchment is calculated here as a function of the catchment area A, as k = 0.43A^0.324^ (h), where A is in km^2^^36^. The rainfall partitioning into infiltration and runoff components has been calculated using the SCS-CN method^37^. We used an average CN at catchment scale (in average moisture conditions, i.e., Antecedent Soil Moisture Condition Class II) obtained from the world map of CN developed in^38^. The CN values obtained are in the range [65, 83]. The precipitation dynamics is described by a Poisson Rectangular Pulses model (^39^, Table F.2). Estimates of intensity (*λ*_I_, h/mm) and duration (*λ*_W_, h^−1^) parameters of the Poisson Rectangular Pulses model are available in 75 rain gauges within continental United States, given in Table F.2,^39^. Of this information, we considered 36 stations, which are representative of 53 catchments (Fig. S16). We considered each rain gauge representative of a circle with 150 km radius. If this area is within a catchment, or this area covered at least 70% of the catchment area, we considered the rain gauge as a representative gauge for the catchment. Table F.2 in^39^ provides the monthly estimates of *λ*_I_ and *λ*_W_ parameters as well as the monthly estimate of the Poissonian chronology. Assuming the intensity and the duration of precipitation events, independent each other, using Monte Carlo technique, we generated 100 yrs of synthetic time series of rainfall events, using the monthly estimates of *λ*_I_ and *λ*_W_ parameters and a number of events for each month equal to the monthly estimate of the Poissonian chronology. In particular, in order to account for the seasonality of flood events in U.S.^40^, we have restricted the analysis to six months: January, February, September-December, filtering out the period of the year, where the snowmelt is principal driver of flood events. Then, we have calculated the synthetic hydrographs from the generated rainfall events. Since the linear reservoir generates response with infinite duration, we used a threshold equal to the 10th percentile of daily discharge in order to identify the duration and the volume of the flood event.

**Figure 2:**
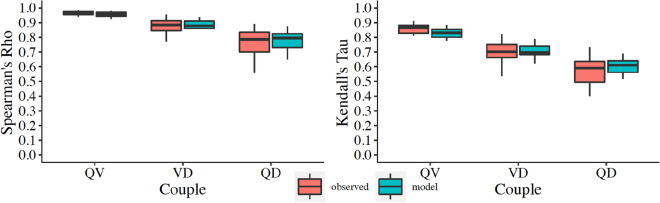
Comparison between observed (using 90th percentile of daily discharge as threshold) and simulated pairwise dependencies in terms of boxplots, for U.S subdataset. Spearman’s rho is in the left panel, and the Kendall’s tau in the right panel.

Figure 2 shows the comparison between observed (using the 90th percentile as threshold) and simulated pairwise dependencies of flood events (in the left panel for the Spearman’s rho, and in the right panel for the Kendall’s tau) relatively to six months of the year: January, February, September-December. For sake of clarity, in Figs. S17–S18, we have reported similar comparisons, but using respectively the 80th and 95th percentiles. From Fig. 2 (as well as Fig. S18), it is possible to see that the rainfall-runoff model, even if crude, is able to reproduce the observed pairwise dependencies, quite well. It is possible to see some differences in Fig. S17 for the 80th percentile, suggesting probably that a threshold equal to 90th or 95th percentile of daily discharge works better than the 80th percentile to identify the catchment response to rainfall forcing.

**Figure 3:**
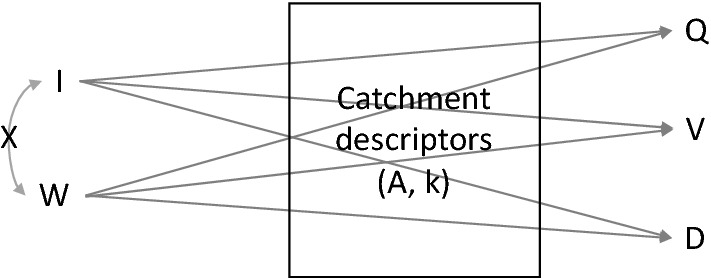
Path diagram between meteorological (exogenous) variables I and W, and the flood (endogenous) variables: Q, V, D. I and W can be dependent (represented by a curved line with arrowheads at each end) or independent (represented with the symbol × on the curved line) each other. The connection between each meteorological variable and each flood variable is filtered by the catchment descriptors (A, k, in this case) and represented by an arrow that goes from each cause to the effect.

Once realized that the conceptual rainfall-runoff model is able to reproduce the observed pairwise dependencies, of rainfall-driven flood events, we have investigated the origin of pairwise dependencies between the flood event variables in the conceptual model, as a proxy information of the natural flood events, rainfall-driven. Following the terminology used by^41^, the meteorological variables, I precipitation intensity and W precipitation duration, which are the causes, are denominated *exogenus*, or independent, variables. For simplicity, we are assuming here I and W independent each other, even if other studies have shown a dependence between these two^42,43^, in addition, we assumed the catchment as impervious. These assumptions will not alter the conclusions of the study, rather than keep simple the problem under analysis. The flood event variables Q, V, D are denominated *endogenus*, or dependent, variables. The functional relations, *f*(·), connecting each dependent variable to the independent ones are the following

1 $$\left\{ {\begin{array}{*{20}l} {{\text{Q}} = f_{{\text{Q}}} \left( {{\text{I}},{\text{W}};{{\varvec{\Psi}}}} \right) = {\text{c}} \cdot {\text{I}} \cdot {\text{A}} \cdot \left( {1 - exp\left( { - \frac{W}{{\text{k}}}} \right)} \right) - {\text{q}}_{{\text{T}}} } \hfill \\ {{\text{V}} = f_{V} \left( {{\text{I}},{\text{W}};{{\varvec{\Psi}}}} \right) = \left( {{\text{c}} \cdot 3600} \right){\text{I}} \cdot {\text{A}} \cdot {\text{W}} - \mathop \smallint \limits_{0}^{ + \infty } {\text{q}}^{*} \left( {\text{t}} \right)dt} \hfill \\ {{\text{D}} = f_{{\text{D}}} \left( {{\text{I}},{\text{W}};{{\varvec{\Psi}}}} \right) = t_{2} - t_{1} = W + k \cdot \left[ {ln\left( {\frac{{{\text{c}} \cdot {\text{I}} \cdot {\text{A}} - {\text{q}}_{{\text{T}}} }}{{{\text{q}}_{{\text{T}}} }}} \right) + ln\left( {1 - exp\left( { - \frac{{\text{W}}}{{\text{k}}}} \right)} \right)} \right]} \hfill \\ \end{array} } \right.$$

where **Ψ** is the vector of parameters representing the catchment (which here are only two: the area A, and reservoir constant k, but in general could include more others like for example the curve number CN). c is a conversion factor (c = 1/3.6), being Q in m^3^/s, I in mm/h, W,D,k in h, V in m^3^, and A in km^2^. Equation (1) is the particular case, for an impervious catchment, of Eq. (10) valid for a pervious catchment, and given in the Materials and Methods Section. q_T_ is the threshold used on the discharge q(t), in the second equation of Eq. (1), the integrand term q*(t) is q*(t) = q(t) if q(t) < q_T_, otherwise q*(t) = q_T_, and t_2_ and t_1_ are the instant times having q(t_1_) = q(t_2_) = q_T_. Figure 3 describes graphically Eq. (1) reporting the connections between the dependent and independent variables.

Let’s use Eq. (1) to investigate the pairwise dependencies between the flood event variables.

Let’s consider for simplicity q_T_ = 0, and fix the catchment parameters (A, k). Keeping fixed W, and increasing (decreasing) I, it increases (decreases) linearly both Q and V, leading to a perfect positive dependence between Q and V, and thus to a unitary Spearman’s rho and Kendall’s tau [according to Eq. (4)]. Vice-versa, keeping fixed I, and increasing (decreasing) W, it increases (decreases) both Q and V. V increases (decreases) linearly, while Q increases(decreases) non-linearly with an exponential function, however, we have again unitary Spearman’s rho and Kendall’s tau [according to Eq. (5)].

In general, both W, and I vary (independently or dependently one from the other). Using in Eq. (1), the Taylor approximation at the first order of the term $\left( {1 - exp\left( { - \frac{{\text{W}}}{{\text{k}}}} \right)} \right)$ of Q, i.e., $\left( {1 - exp\left( { - \frac{{\text{W}}}{{\text{k}}}} \right)} \right) \approx \frac{{\text{W}}}{{\text{k}}}$, we can conclude again that Spearman’s rho and Kendall’s tau are both equal to 1 [according to Eq. (8)]. Note that this result is independent by the presence (or not) of dependence between I and W, and in addition, it is independent by the catchment area A, in agreement with what it is found in Fig. 1, i.e., the catchment area does not influence the dependence between Q and V.

Without the Taylor approximation at the first order, using generated pairs (I, W) considering I and W independent, we found (numerically) that the values of Spearman’s rho and Kendall’s tau, are still positive, high (say > 0.9), but not equal to 1.

These reasonings are in agreement with the high values of Q–V dependence (both in terms of Spearman’s rho, and Kendall’s tau) observed in the worldwide analysis.

Considering q_T_ ≠ 0, and reasoning in terms of D, from Eq. (1), we can see that the link between D and W is not explicit. However, for W →  + ∞, D increases linearly with W. Increasing (decreasing) I, D increases (decreases) logarithmically with I. Thus, we can infer that also Q–D and V–D dependencies are positive. We found also that the strength of the dependence between two variables depends by the similarity of the functional relation they have with the independent variables. The Q–V dependence exhibited the highest values because Q and V have both linear relation with I and similarly a linear relation with W for V and approximately for Q. V–D and Q–D dependence values resulted smaller respect to those relative to Q–V, with values of V–D dependence greater than those relative to Q–D dependence, this because V and D have both asymptotically a linear relation with W but different functional relation with I, while Q and D exhibit different function with both I and W. As last note, we want to observe that the pairwise dependence between the flood event variables exists even if the two exogenous variables are independent each other. The effect of dependence between I and W can strengthen or weaken the pairwise dependence between the flood event variables. According to some preliminary simulations, we found that positive association between I and W strengthens the pairwise dependence between the flood event variables, while negative association weakens these associations.

## Discussion

The analysis of a worldwide database of daily discharge and the calculation of the pairwise statistical dependence, via the Kendall’s tau and Spearman’s rho, between peak, volume, and duration of flood events, selected with a threshold-based approach and a criterion of temporal independence of events, gave an indication of the variability spectrum of flood event dependencies. This analysis does not distinguish among the different flood generating mechanisms (rainfall-driven, snow-melt, rain-on-snow, glacier-melt floods), and thus the results must be considered in this respect, evenif high values of the threshold (namely, 80th, 90th, 95th percentile) could allow referring to the rainfall-driven component of flood events. Thus, further dependence analyses, conditioned by different flood generating mechanisms could permit to appreciate the differences due to the different flood drivers. In addition, note that, with the Kendall’s tau and Spearman’s rho, we have assessed the general dependence between each pair of variables, while for extreme values purposes, one could be interested in evaluating the dependence in correspondence of the tails, and using the upper-tail or the lower tail dependence coefficients^28^.

Then, a conceptual rainfall-runoff model has been considered to reproduce the observed pairwise dependencies, relatively to the case of rainfall-driven flood events and for the subdataset of continental United States. The model is a crude conceptualization of rainfall-runoff processes. Specifically, we neglected the contributions to discharge due to snow, ice melting and groundwater flow, which will provide other elements to complicate the puzzle. We used such crude conceptualization deliberately, in order to keep analitically tractabale the problem, and understand the mechanistic connections between inputs and outputs. The comparison between simulated dependencies and observed ones at different thresholds indicates that a threshold equal to 90th or 95th percentile works better than the 80th percentile, to identify the catchment response to rainfall forcing.

We used a model-dependent realism approach and accordingly we investigated the origin of the dependencies between the event variables (Q, V, D), specifically for the case of rainfall-driven flood events, and for impervious catchments. For these, we realized that the origin of the dependencies between the flood event variables is spurious, not causal, it comes from the fact that all the three variables (Q, V, D) are caused/generated by the same two exogenus variables (I, W). This fact can be summarized recalling the Reichenbach's Common Cause Principle, also known as “no correlation without causation”, reported in^41^. It states that, if any two variables are dependent, then one is a cause of the other, or there is a third variable causing both. Here, we have shown that this is the second case, i.e., in the (Q, V) dependence, as well as in the (Q, D) and (V, D) dependencies, there are third variables (namely, I and W) which cause the dependence.

This result sheds light on the compound nature of flood events rainfall-driven, where the dependence between flood event characteristics (Q, V, D) emerges as a consequence of the relation of such characteristics to the rainfall input variables (I, W) that control the hydrographs. In addition, this result puts light also on the multivariate modeling of flood event characteristics (Q, V, D) stating that there is not a causal priority among these variables to be used in conditional analysis and modeling.

Note that disentangling the origin of pairwise dependence between V, Q, and D, would be difficult (if not impossible) without a model-dependent realism approach, and dealing only with data-based analyses.

The approach based on the model-dependent realism is quite general and could be extended in different directions: (1) including, in the hydrological model, other mechanisms generating flood events like snow-melt, rain-on-snow, glacier-melt floods^44–46^, (2) considering other hydrological models, with more realistic assumptions (^47–50^, among others), and comparing these with the simplified model used here; (3) investigating other hydrological problems in order to study the typology of dependence between the variables, and the existence or not of causality.

## Materials and methods

### Dataset and flood events sampling

Here, we used the Global Runoff Database (GRDC) consisting in 4638 stations collecting the daily discharge within the period 1812–2014 (available at the website:

https://www.bafg.de/GRDC/EN/01_GRDC/13_dtbse/database_node.html). The time series have a number of years varying in the range 25—198 years, with the 1^st^ quartile equal to 39 years, a median of 50 years, and the 3^rd^ quartile equal to 76 years, covering 80 countries in the world. 15 countries contain only one station and the United States with 929 stations has the utmost number of stations in the dataset. The range of the catchment area is between 0.07 km^2^ (in Finland) to 4,680,000 km^2^ (in Brazil, the Amazon river). Since the temporal resolution of data is one day, we have not considered in the analysis small catchments with temporal dynamics within the day, roughly saying catchments with an area smaller than 500 km^2^.

In literature there are several methods used to select a flood event (e.g.,^51–54^). Among these, here, we have chosen a simple method based on a discharge threshold, a fixed percentile of daily discharge, also adopted in^18,32,51,53,55–57^. The beginning and end of a flood event are respectively the first and last value, when the discharge is greater or equal the threshold. The flood duration D is the time during which the discharge is greater or equal the threshold, the flood volume V is the volume of the above the threshold, the flood peak Q is the maximum value of daily discharge occurred within the event duration subtracted the threshold. Thus, each part of hydrograph above the threshold represents a flood event. In order to ensure that the extracted flood events are temporal independent, we considered a criterion of temporal independence of flood events based on the minimum time, T*, between the end of an event and the beginning of the next one. In particular, T* was set to 5 days for catchments with area < 45,000 km^2^; 10 days for catchments with area between 45,000 and 100,000 km^2^; and 20 days for catchments with area > 100,000 km^2^; slighting modifying^58^, where this criterion was applied to the time between the peaks of the two successive events. In the case two events resulted dependent, we discharged the one associated to the minor peak. Fig. S1 gives a sketch of the flood events selection. We used a threshold-conditioned, or threshold-based definition of flood event, basically for two reasons: (1) flexibility: the threshold based on a fixed percentile of daily discharge can account for the local condition of discharge and work in different contexts and stations as requested here; (2) physical meaning of the threshold. It can be the bankfull discharge^57^, or the value exceeding the embankment crest^55^, so the threshold-based flood event can represent the dangerous part of hydrograph producing problems and damages. Here we have considered as threshold: 80th, 90th, and 95th percentile of daily discharge (also used in^18^), thus high values of daily discharge. We made this choice because, we intend to consider only the catchment response to rainfall forcing, or the rainfall-driven component of flood events, and leave out the processes which produce the base flow (i.e., snow and ice melting, subsurface or groundwater flow). Figs. S2–S4 provide the location of the stations considered, with and without at least 40 flood events, for the different thresholds, respectively 80th, 90th, 95th percentile.

The extracted time series of Q, V and D, have the problem of ties (i.e., repeated values), due to the daily time resolution and 1 m^3^/s resolution for the discharge. Let x be the true value of the variable X under investigation and let Δ be the resolution adopted. x belongs to a unique interval [Δk_X_, Δ(k_X_ + 1)] for a suitable integer k_X_, but the true value x is incorrectly stored in the database as, say, x* = Δk_X_. All the occurrences falling into the interval are wrongly recorded as x* (ties). The percentage of ties for D is in the range [7%, 99%], for Q is in the range [0%, 96%], and for V is in the range [0%, 92%], depending on the site, and threshold considered. These ties could impact in the calculation of pairwise dependencies. For this, we have used a randomization technique, also known as “jittering”, to remove repeated values and provide the natural granularity of the observed phenomenon, following^34^. This technique consists in substituting repeated values x^*^ by transforming each available ${\text{x}}_{{\text{i}}}^{*}$ as $\tilde{x}_{i} = x_{i}^{*} + \Delta \cdot u_{i}$ where *u*_*i*_’s are i.i.d. r.v.’s Uniform over [0, 1]. We used a Uniform distribution to randomize the ties, because it is an “noninformative” distribution, and in order to avoid unnecessary assumptions associated to this randomization.

The data of each variable, selected at each threshold, have been checked against the autocorrelation, and non-stationarities via three tests^59^: the Pettitt test for the presence of changing points, the Mann–Kendall test for the presence of monotonic trends, and the test on the presence of autocorrelation in order to check the stochastic independence between events. For all tests, we considered a 1% significance level. For each site and each threshold, if all variables (Q, V and D) passed all the three tests, we considered that site for further analyses with respect to that threshold, otherwise we have removed it. Thus, for the 80th, 90th, and 95th percentile, we considered respectively 1310, 1602 and 1922 stations, corresponding respectively to about 28%, 35% and 41% of the dataset. Increasing the level of threshold, the probability of rejection the tests as well dependencies, is low respect to low-level threshold ^35^. Then, we focused the attention to the stations with at least 40 data, for each threshold, in order to have enough data to estimate the measures of dependence. Thus, for the 80th, 90th, and 95th percentile, we considered respectively 1140, 1316 and 1411 stations, corresponding respectively to about 25%, 28% and 30% of the dataset.

### Dependence measures

Here we used two measures of association, namely the Kendall’s tau (τ) and the Spearman’s rho (ρ), which provide a form of dependence, known as association^28^. These are two non-parametric rank-based measures, which always exist. Let (X, Y) a vector two continuous random variables. Let (X_1_, Y_1_) and (X_2_, Y_2_) be independent and identically distributed random vectors, with X_1_ and X_2_ having the same distribution of X and similarly, Y_1_ and Y_2_ having the same distribution of Y, and having the same joint distribution of (X, Y). The Kendall’s tau between X and Y is defined as

2 $$\tau_{{{\text{XY}}}} = {\text{P}}\left[ {\left( {{\text{X}}_{1} - {\text{X}}_{2} } \right)\left( {{\text{Y}}_{1} - {\text{Y}}_{2} } \right) > 0} \right] - {\text{P}}\left[ {\left( {{\text{X}}_{1} - {\text{X}}_{2} } \right)\left( {{\text{Y}}_{1} - {\text{Y}}_{2} } \right) < 0} \right]$$

where the first term on the right side is the probability of concordance, while the second one is the probability of discordance.

Similarly, to the Kendall’s tau, also the Spearman’s rho is a based on the probability of concordance and the probability of discordance. Let (X_1_, Y_1_), (X_2_, Y_2_), and (X_3_, Y_3_) be three independent random vectors with a common joint distribution function, equal to the one of (X, Y). The Spearman’s rho between X and Y is defined as

3 $$\uprho _{{{\text{XY}}}} = 3{\text{P}}\left[ {\left( {{\text{X}}_{1} - {\text{X}}_{2} } \right)\left( {{\text{Y}}_{1} - {\text{Y}}_{3} } \right) > 0} \right] - {\text{P}}\left[ {\left( {{\text{X}}_{1} - {\text{X}}_{2} } \right)\left( {{\text{Y}}_{1} - {\text{Y}}_{3} } \right) < 0} \right]$$

i.e., the probability of concordance minus the probability of discordance for the two vectors (X_1_, Y_1_) and (X_2_, Y_3_).

Both the Kendall’s tau and the Spearman’s rho vary in the range -1 and 1. If the variables are independent each other, the Kendall’s tau and the Spearman’s rho are equal to 0.

Let us consider now two variables X′ and Y′ transformations of the variables X, Y and see how change the correspondent two measures of association, which will be both indicated with the symbol *δ*.

*Case 1a*. X′ = aX, Y′ = bX (i.e., linear transformations of a single variable), where a and b are two constants, the Kendall’s tau and the Spearman’s rho of X′ and Y′ are

4 $$\delta_{{{\text{X}}^{\prime } {\text{Y}}^{\prime } }} = \pm 1$$

where the sign (+ or −) is depending on the sign of the product a·b (+ or −).

*Case 1b*. X′ = g(X), Y′ = h(X), where g(·) and h(·) are two strictly monotone (both increasing, or both decreasing) functions of the variable X. The Kendall’s tau and the Spearman’s rho of X′ and Y′ are

5 $$\delta_{{{\text{X}}^{{\prime }} {\text{Y}}^{{\prime }} }} = + 1$$

*Case 2a*. X′ = aX, Y′ = bY (i.e., linear transformations of two variables). The Kendall’s tau and the Spearman’s rho of X′ and Y′ are:

6 $$\delta_{{{\text{X}}^{{\prime }} {\text{Y}}^{{\prime }} }} = \pm \delta_{{{\text{XY}}}}$$

the sign (+ or −) is depending on the sign of the product a·b (+ or −).

*Case 2b*. X′ = g(X), Y′ = h(Y), where g(·) and h(·) are two strictly monotone (both increasing, or both decreasing) functions respectively of X and Y. The Kendall’s tau and the Spearman’s rho of X′ and Y′ are invariant respect to those of X and Y:

7 $$\delta_{{{\text{X}}^{{\prime }} {\text{Y}}^{{\prime }} }} = \delta_{{{\text{XY}}}}$$

*Case 3a*. X′ = aXY, Y′ = bXY (i.e. both X′ and Y′ are directly proportional to the product XY), where a and b are two constants, the Kendall’s tau and the Spearman’s rho of X′ and Y′ are

8 $$\delta_{{{\text{X}}^{{\prime }} {\text{Y}}^{{\prime }} }} = \pm 1$$

where again the sign (+ or −) is depending on the sign of the product a·b (+ or −).

*Case 3b*. X′ = g_2_(X, Y), Y′ = h_2_(X, Y), where g_2_(·) and h_2_(·) are two functions of both X and Y. This is the general case. The dependence between X′ and Y′ can be determined numerically with Montecarlo simulations.

We used Kendall’s tau, also the Spearman’s rho to evaluate the dependence between each pair of flood event characteristics, for each threshold level. For the estimation of the dependence measures, we considered the minimum sample size of 40 events. If $\left( {{\text{x}}_{1} {\text{y}}_{1} } \right) \left( {{\text{x}}_{2} {\text{y}}_{2} } \right) \ldots \left( {{\text{x}}_{{\text{n}}} {\text{y}}_{{\text{n}}} } \right)$ be a sample from the vector (X, Y). The sample version of the Kendall’s tau is ${\text{t}} = \frac{{{\text{c}} - {\text{d}}}}{{{\text{c}} + {\text{d}}}}$^28^, where c is the number of concordant pairs, i.e. the couple satisfying the relation (x_i_ − x_j_)(y_i_ − y_j_) > 0, while d is the number of discordand pairs, i.e. the couple satisfying the relation (x_i_ − x_j_)(y_i_ − y_j_) < 0. The sample version of the Spearman’s rho is ${\text{r}} = 1 - \frac{{6\mathop \sum \nolimits_{{{\text{i}} = 1}}^{{\text{n}}} \left( {{\text{R}}_{{\text{i}}} - {\text{S}}_{{\text{i}}} } \right)^{2} }}{{{\text{n}}^{3} - {\text{n}}}}$^28^, where R_i_ = Rank(x_i_), S_i_ = Rank(y_i_), and n is the sample size.

### Hydrological rainfall-runoff model

In order to describe the response of a catchment to a rainfall event, we considered a linear reservoir model, characterized by a storage constant k. This last has been calculated as a function of the catchment area A, as k = 0.43A^0.324^ (h), with A in km^2^^36^. The precipitation dynamics has been described by a Poisson Rectangular Pulses model^39^, i.e. a sequence of rectangular rainfall events, having a Poissonian chronology (with parameter Λ), where the duration W (h) is exponential, with parameter λ_W_ (h^−1^) and the mean rainfall intensity I (mm/h), constant over the duration of rainfall event and catchment area, is still exponential, with parameter λ_I_ (h/mm). Monthly estimates of the parameter λ_W_, λ_I_, and Λ are available in 75 stations within continental United States, given in Table F.2,^39^. The rainfall partitioning into infiltration and runoff components has been calculated using the SCS-CN method^37^. We used an average CN at catchment scale (in average moisture conditions, i.e., Antecedent Soil Moisture Condition Class II) obtained from the world map of CN developed in^38^. We calculated the effective rainfall intensity (or runoff) I^*^ from I as ${\text{I}}^{*} = \left( {1/{\text{W}}^{*} } \right)\left( {{\text{I}} \cdot {\text{W}} - {\text{I}}_{{\text{a}}} } \right)^{2} /\left( {{\text{I}} \cdot {\text{W}} - {\text{I}}_{{\text{a}}} + {\text{S}}} \right)$ if ${\text{I}} \cdot {\text{W}} > {\text{I}}_{{\text{a}}}$, otherwise I^*^ = 0, where I_a_ (mm) is the initial abstraction, evaluated as I_a_ = 0.2S, S (mm) is the maximum soil potential retention, calculated as ${\text{S}} = 254\left( {\frac{100}{{{\text{CN}}}} - 1} \right)$, W^*^ is the effective rainfall duration, calculated as ${\text{W}}^{*} = {\text{W}} - {\text{I}}_{{\text{a}}} /{\text{I}}$ if ${\text{W}} > {\text{I}}_{{\text{a}}} /{\text{I}}$, otherwise W^*^ = 0. In the case of impervious catchments [used in Eq. (1)], CN = 100, S = 0, W^*^ = W and I^*^ = I.

The flood hydrograph q(t), in m^3^/s, is described as

9 $${\text{q}}\left( {\text{t}} \right) = \left\{ {\begin{array}{*{20}l} {{\text{c}} \cdot {\text{I}}^{*} \cdot {\text{A}} \cdot \left( {1 - exp\left( { - \frac{{\text{t}}}{{\text{k}}}} \right)} \right)} \hfill & {\quad {\text{t}} \le {\text{W}}^{*} } \hfill \\ {{\text{Q}} \cdot exp\left( { - \frac{{\left( {{\text{t}} - {\text{W}}^{*} } \right)}}{{\text{k}}}} \right)} \hfill & {\quad {\text{t}} > {\text{W}}^{*} } \hfill \\ \end{array} } \right.$$

where c is a conversion factor (c = 1/3.6), Q is the flood peak given by ${ }Q = {\text{c}} \cdot {\text{I}}^{*} \cdot {\text{A}} \cdot \left( {1 - exp\left( { - \frac{{{\text{W}}^{*} }}{{\text{k}}}} \right)} \right)$, the flood volume (in m^3^)${\text{V}} = \left( {{\text{c}} \cdot 3600} \right){\text{I}}^{*} \cdot {\text{A}} \cdot {\text{W}}^{*}$, while the flood duration is infinite. Thus, a threshold q_T_ on the discharge was used in order to identify the duration and the volume of the flood event, say the 10th percentile of daily discharge. Consequently, the flood peak Q, volume V and duration D are respectively

10 $$\left\{ {\begin{array}{*{20}l} {{\text{Q}} = {\text{c}} \cdot {\text{I}}^{*} \cdot {\text{A}} \cdot \left( {1 - exp\left( { - \frac{{{\text{W}}^{*} }}{{\text{k}}}} \right)} \right) - {\text{q}}_{{\text{T}}} } \hfill \\ {{\text{V}} = \left( {{\text{c}} \cdot 3600} \right){\text{I}}^{*} \cdot {\text{A}} \cdot {\text{W}}^{*} - \mathop \smallint \limits_{0}^{ + \infty } {\text{q}}^{*} \left( {\text{t}} \right)dt} \hfill \\ {{\text{D}} = t_{2} - t_{1} = {\text{W}}^{*} + {\text{k}} \cdot \left[ {ln\left( {\frac{{{\text{c}} \cdot {\text{I}}^{*} \cdot {\text{A}} - {\text{q}}_{{\text{T}}} }}{{{\text{q}}_{{\text{T}}} }}} \right) + ln\left( {1 - exp\left( { - \frac{{{\text{W}}^{*} }}{{\text{k}}}} \right)} \right)} \right]} \hfill \\ \end{array} } \right.$$

where the integrand term q*(t) is q*(t) = q(t) if q(t) < q_T_, otherwise q*(t) = q_T_, and t_2_ and t_1_ are the instant times having q(t_1_) = q(t_2_) = q_T_.

## Supplementary Information

Supplementary Information.
