# Supplementary material for: Origin and variability of statistical dependencies between peak, volume, and duration of rainfall-driven flood events
Source: Sci Rep. 2021 Mar 4;11:5182. doi: 10.1038/s41598-021-84664-1 (PMC7970848; doi:10.1038/s41598-021-84664-1)
Supplement: Supplementary file 1 — Supplementary Information. [file 41598_2021_84664_MOESM1_ESM.pdf]

Supplementary Materials – [Short title: Origin and variability of rainfall-driven flood dependencies, L. Rahimi*, C. Deidda*, C. De Michele*]

Table S.1 Kendall's tau and Spearman's rho statistics (min, 1st 2nd 3rd quartile, mean, max) of Q-V, V-D and Q-D association for different thresholds (80th, 90th, and 95th percentile of daily discharge).

		Statistic	Threshold (percentile)		
			80th	90th	95th
Q-V	K. tau	Min	0.610	0.488	0.576
		1st quartile	0.836	0.833	0.831
		2nd quartile	0.856	0.856	0.855
		Mean	0.852	0.849	0.850
		3rd quartile	0.875	0.874	0.877
		Max	0.941	0.930	0.936
	S. rho	Min	0.747	0.649	0.749
		1st quartile	0.959	0.957	0.954
		2nd quartile	0.969	0.968	0.967
		Mean	0.964	0.962	0.961
		3rd quartile	0.976	0.975	0.976
		Max	0.993	0.992	0.993
V-D	K. tau	Min	0.414	0.318	0.174
		1st quartile	0.747	0.706	0.647
		2nd quartile	0.795	0.771	0.729
		Mean	0.781	0.753	0.706
		3rd quartile	0.826	0.812	0.783
		Max	0.921	0.911	0.890
	S. rho	Min	0.586	0.457	0.256
		1st quartile	0.912	0.886	0.839
		2nd quartile	0.941	0.927	0.902
		Mean	0.928	0.909	0.873
		3rd quartile	0.959	0.951	0.935
		Max	0.989	0.987	0.979
Q-D	K. tau	Min	0.309	0.230	0.121
		1st quartile	0.617	0.574	0.522
		2nd quartile	0.666	0.638	0.595
		Mean	0.656	0.625	0.581
		3rd quartile	0.709	0.686	0.653
		Max	0.859	0.827	0.824
	S. rho	Min	0.433	0.341	0.179
		1st quartile	0.811	0.773	0.715
		2nd quartile	0.855	0.832	0.795
		Mean	0.840	0.812	0.769
		3rd quartile	0.890	0.870	0.843
		Max	0.969	0.956	0.946

* Department of Civil and Environmental Engineering, Politecnico di Milano, Milano, Italy.

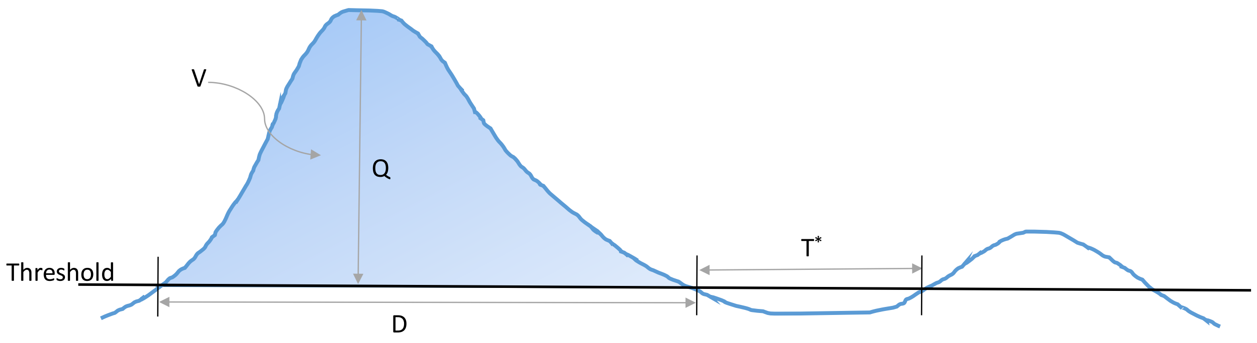

Figure S1. Sketch of flood event selection.

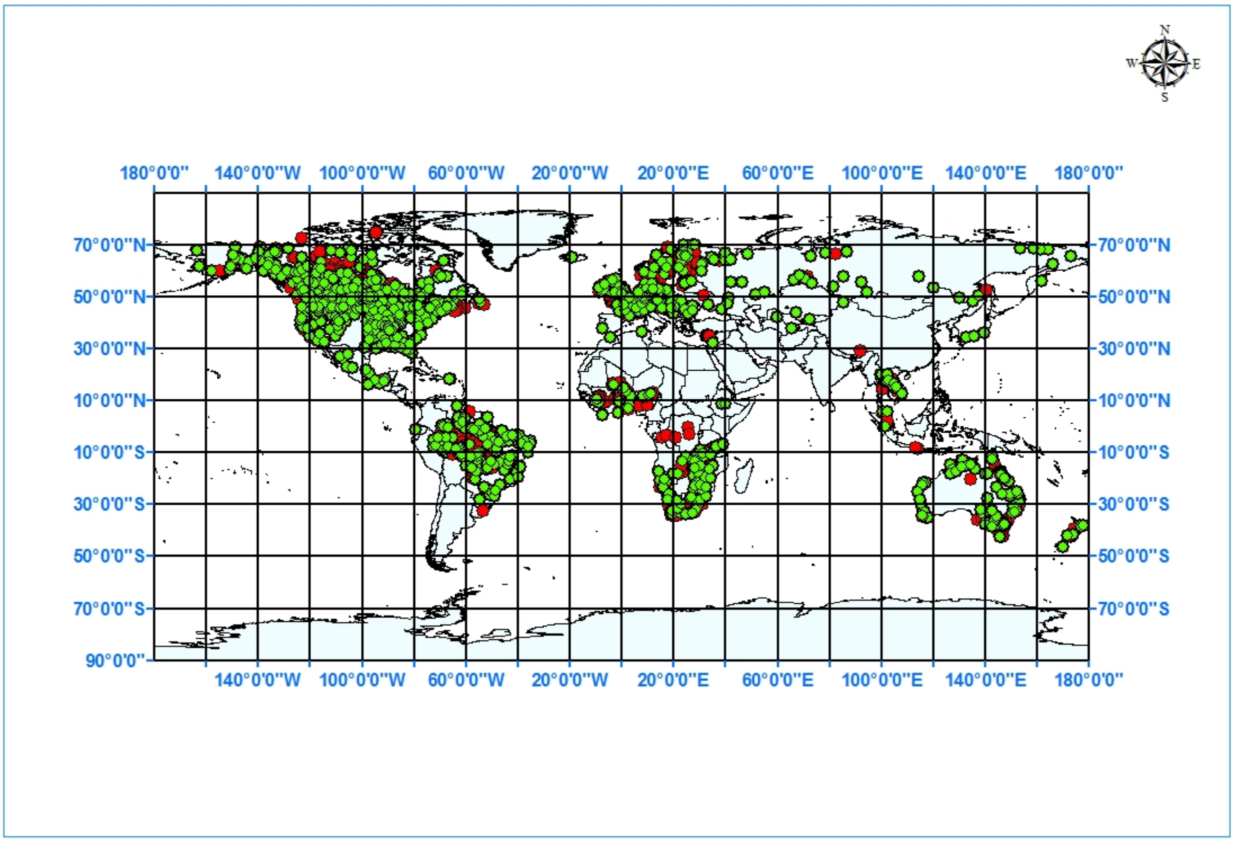

Figure S2. Location of stations, the red points are those associated to a daily discharge threshold equal to the 80th percentile. Green points indicate the stations with a number of events greater than 40.

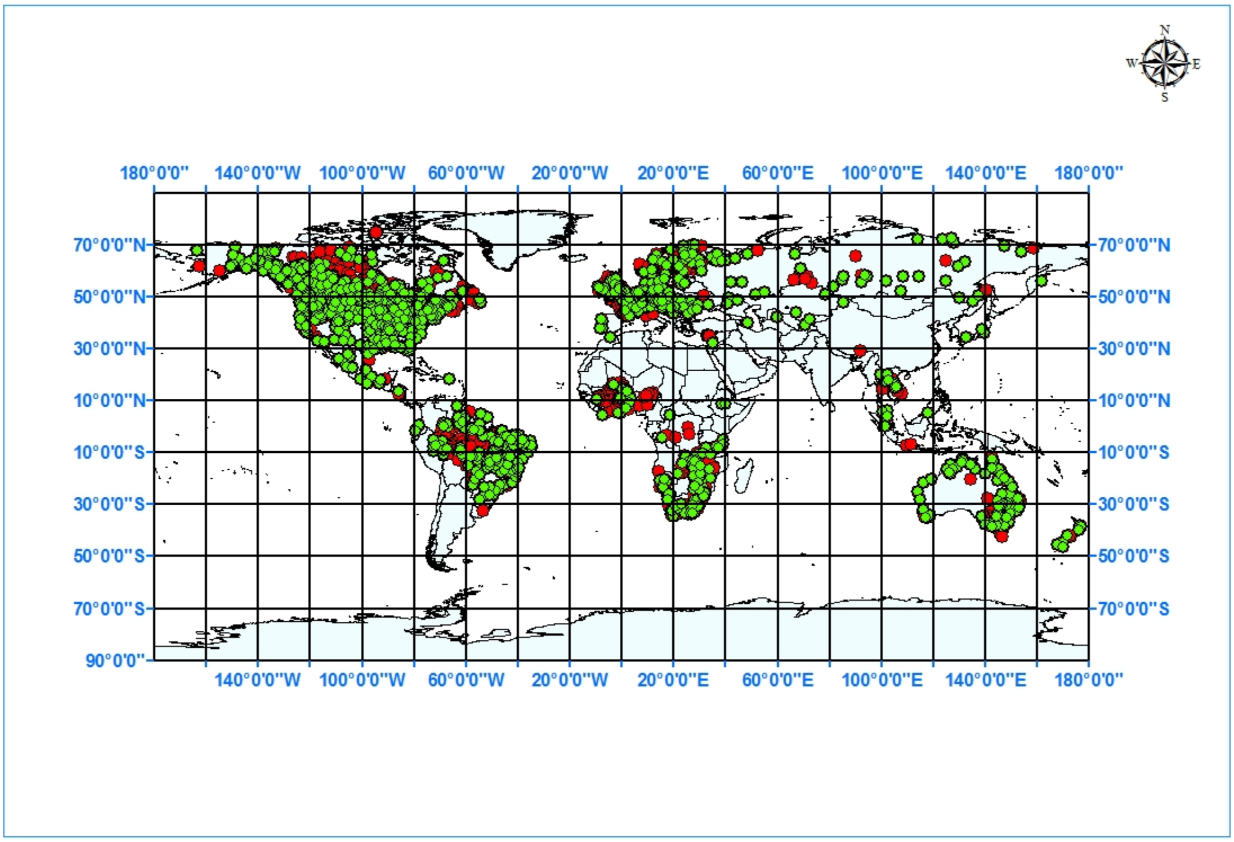

Figure S3. Location of stations, the red points are those associated to a daily discharge threshold equal to the 90th percentile. Green points indicate the stations with a number of events greater than 40.

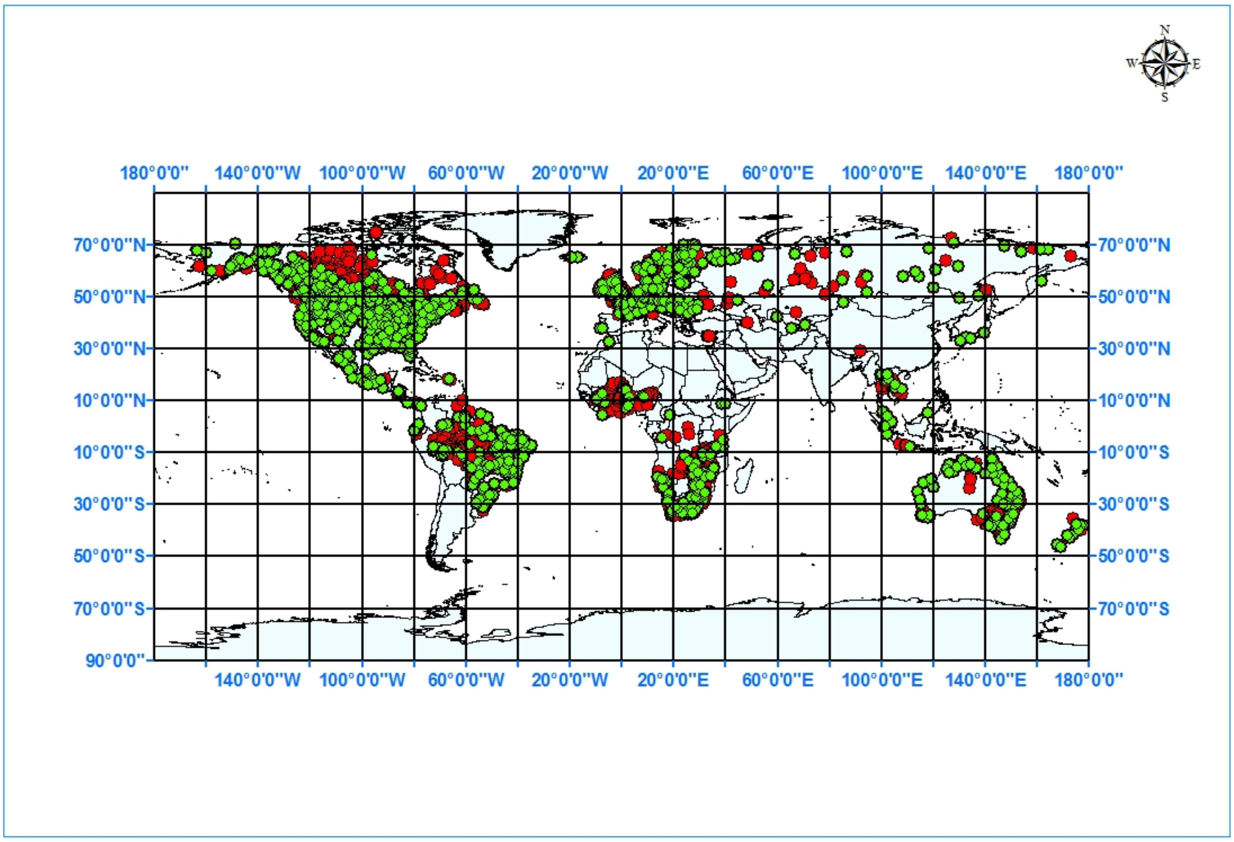

Figure S4. Location of stations, the red points are those associated to a daily discharge threshold equal to the 95th percentile. Green points indicate the stations with a number of events greater than 40.

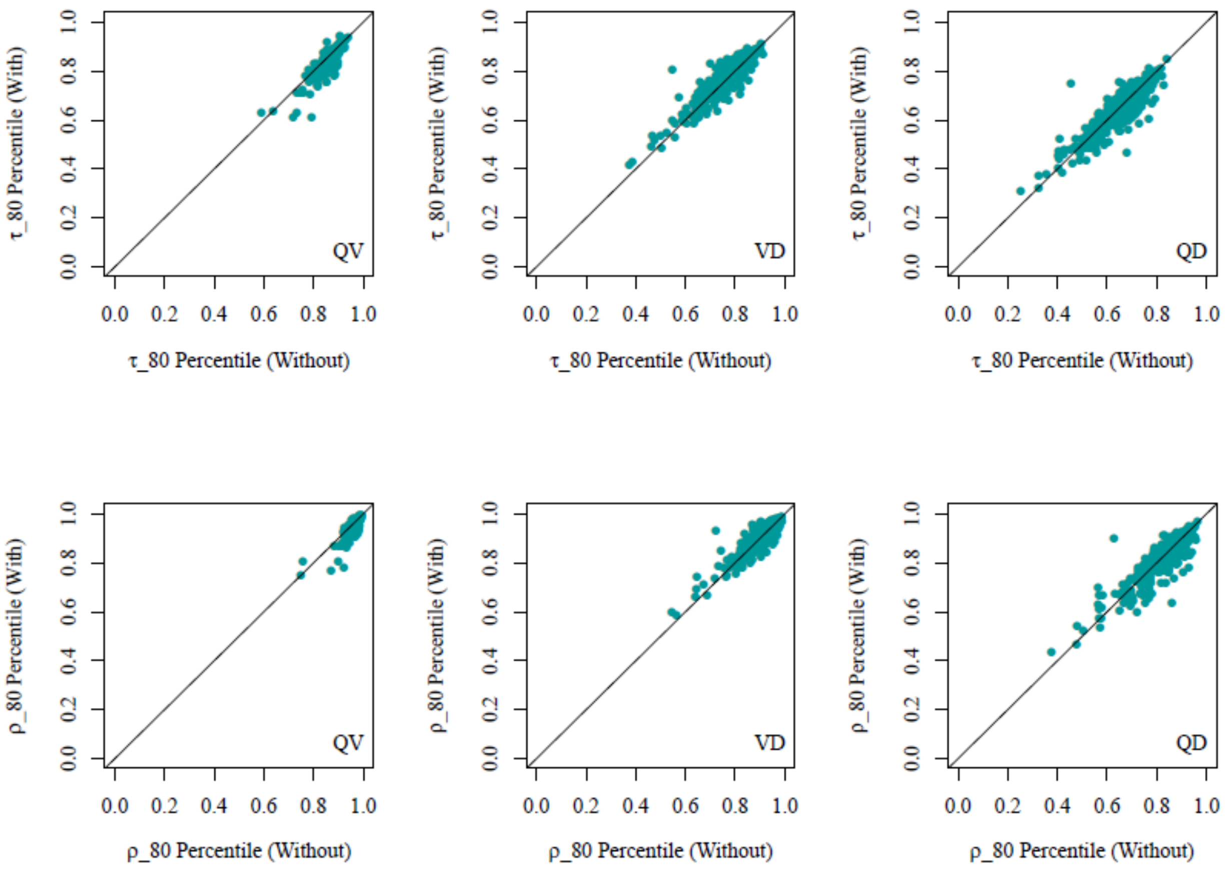

Figure S5. Comparison of the pairwise dependence (Q-V in the left panels, V-D in the central panels, and Q-D in the right panels, using Kendall's tau in the upper panels, and Spearman's rho in the lower panels) considering (with) or not (without) the criterion of temporal independence of flood events, and a daily discharge threshold equal to the 80th percentile.

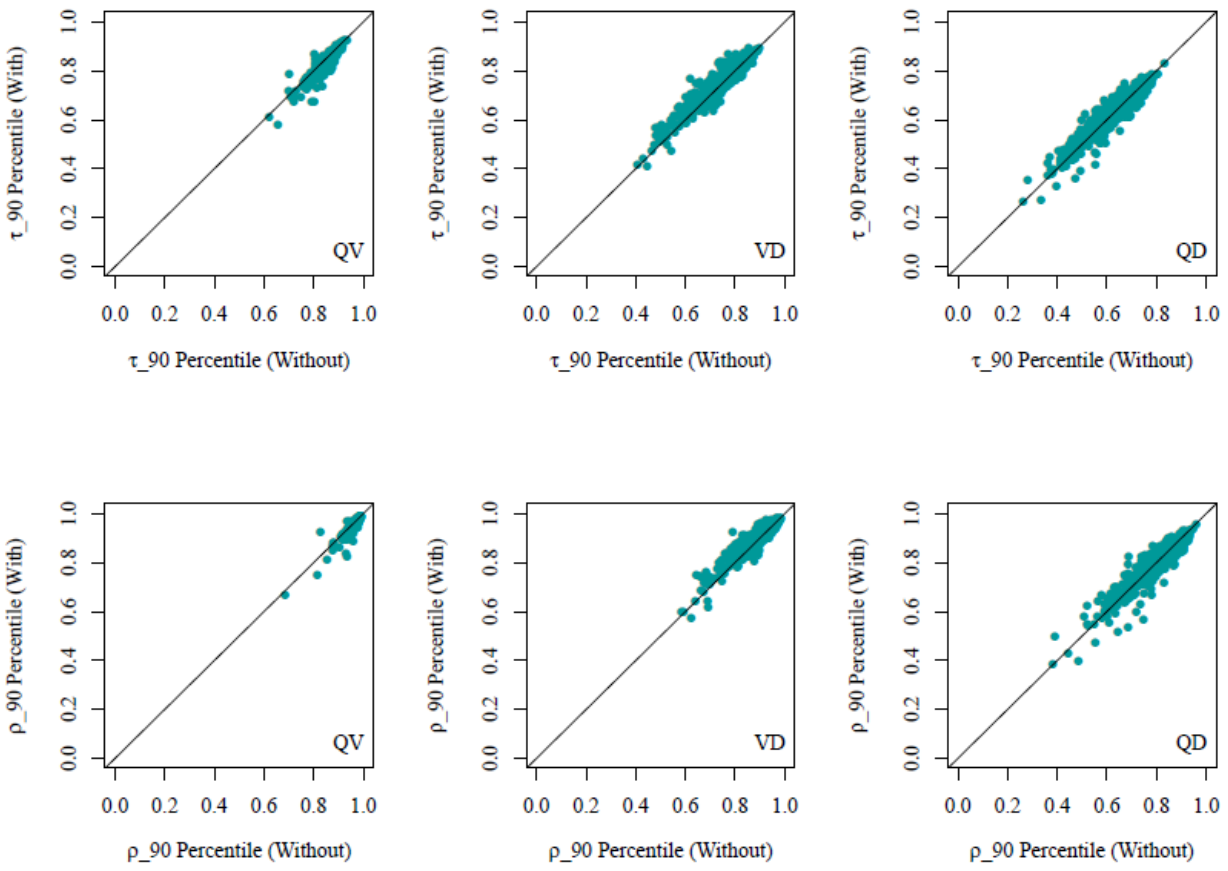

Figure S6. Comparison of the pairwise dependence (Q-V in the left panels, V-D in the central panels, and Q-D in the right panels, using Kendall's tau in the upper panels, and Spearman's rho in the lower panels) considering (with) or not (without) the criterion of temporal independence of flood events, and a daily discharge threshold equal to the 90th percentile.

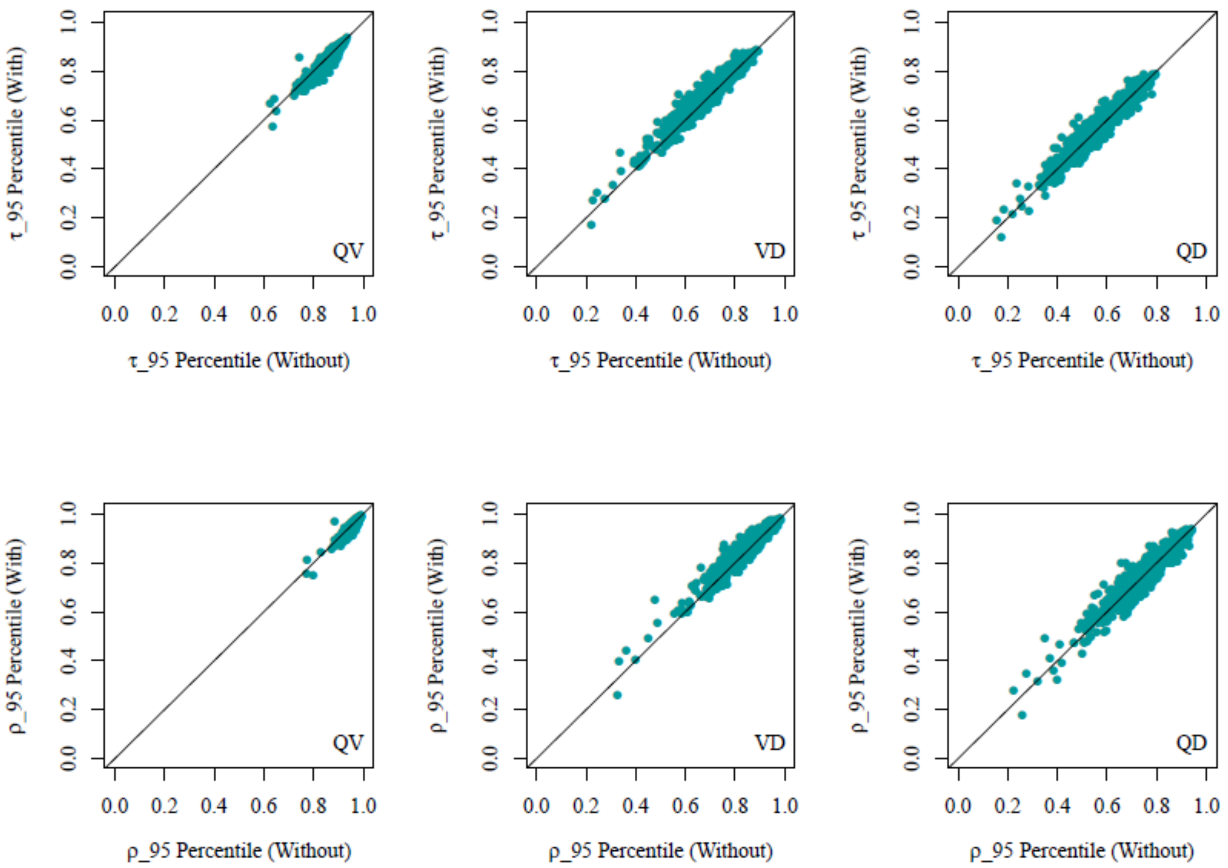

Figure S7. Comparison of the pairwise dependence (Q-V in the left panels, V-D in the central panels, and Q-D in the right panels, using Kendall's tau in the upper panels, and Spearman's rho in the lower panels) considering (with) or not (without) the criterion of temporal independence of flood events, and a daily discharge threshold equal to the 95th percentile.

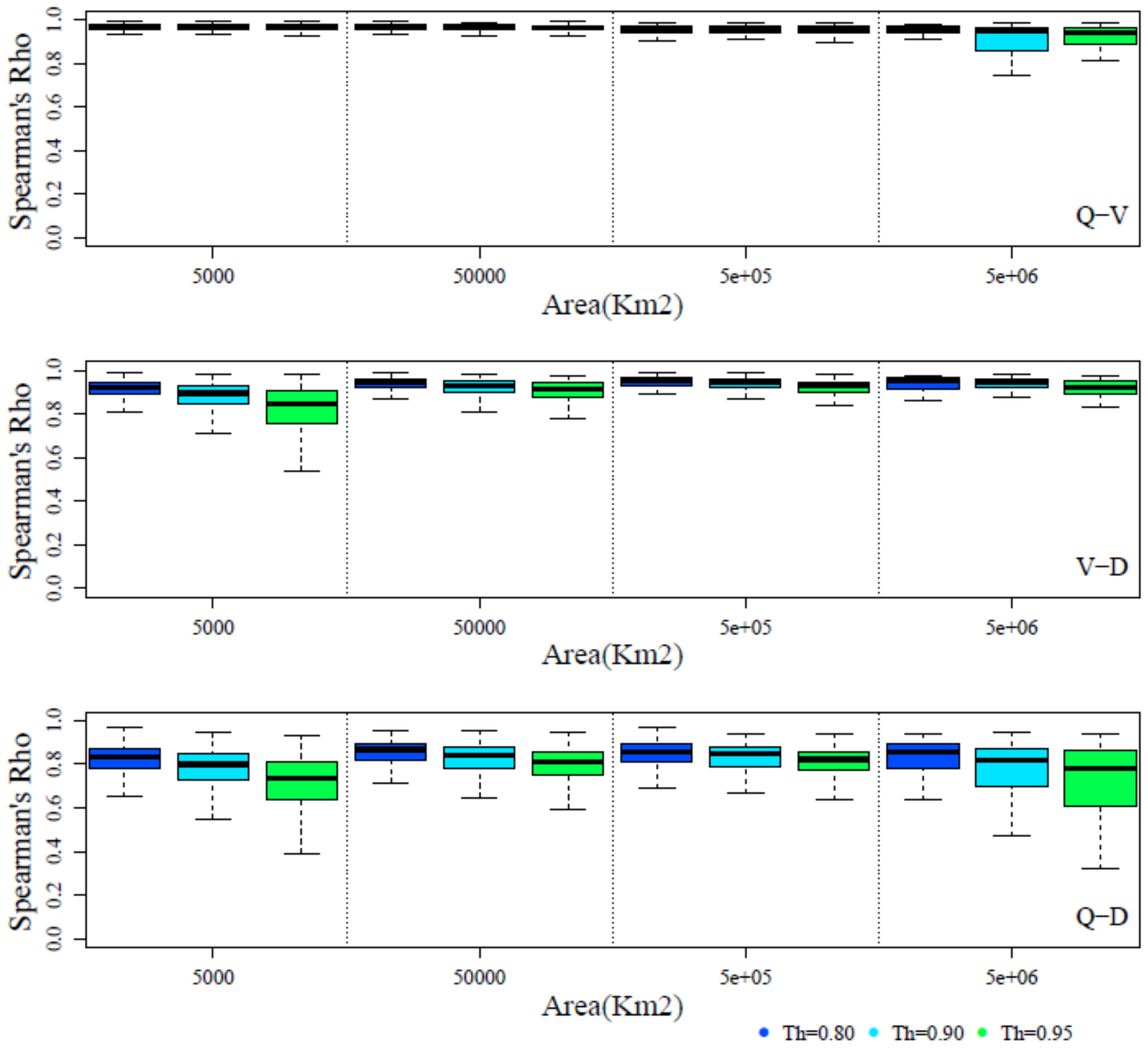

Figure S8. Boxplots of Spearman's rho as function of the catchment area in the all threshold levels considered, for each couple of flood variables (Q-V in the top, V-D in the middle, Q-D in the bottom panel).

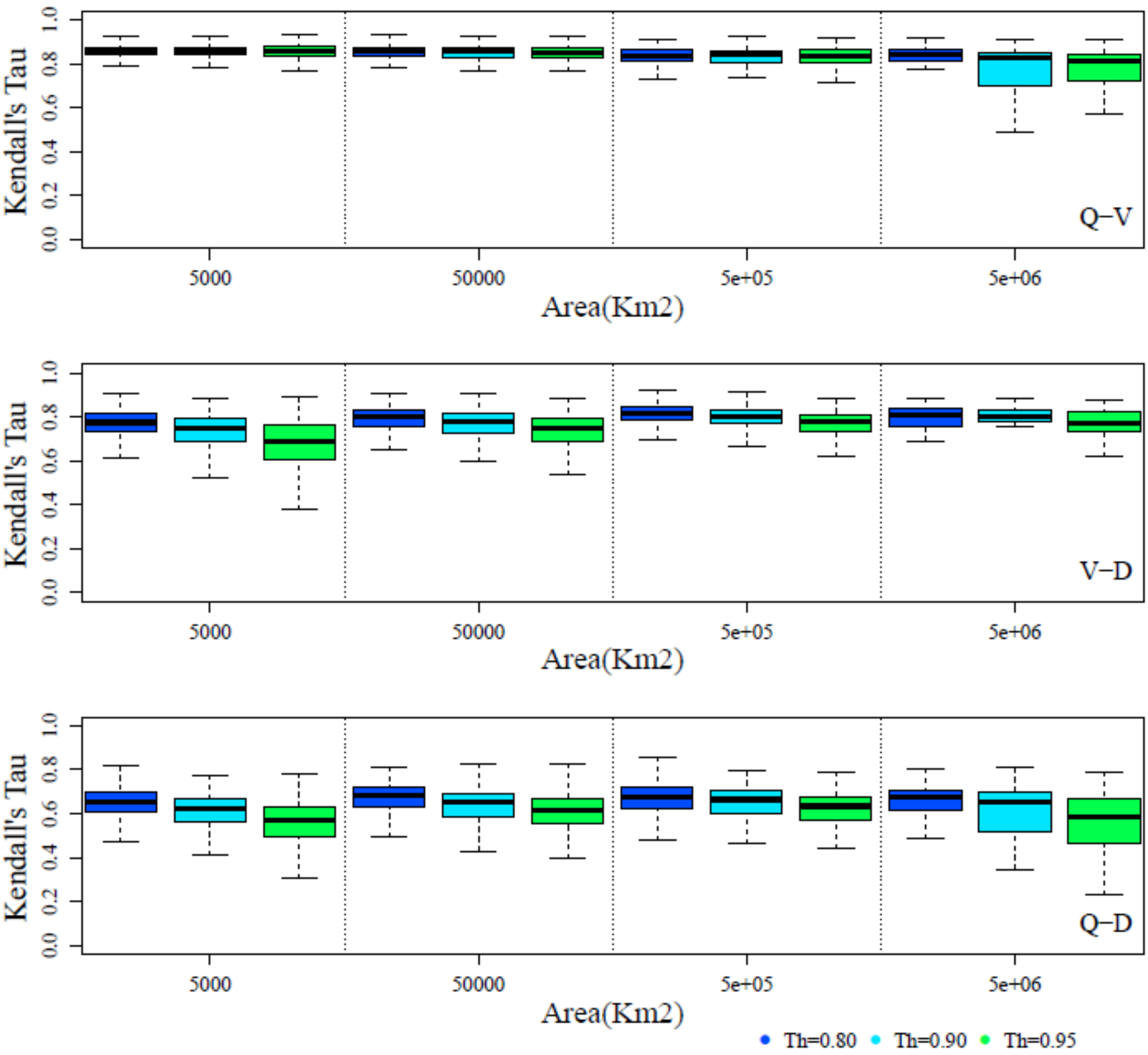

Figure S9. Boxplots of the Kendall's tau as function of the catchment area in the all threshold levels considered, for each couple of flood variables (Q-V in the top, V-D in the middle, V-D in the bottom panel).

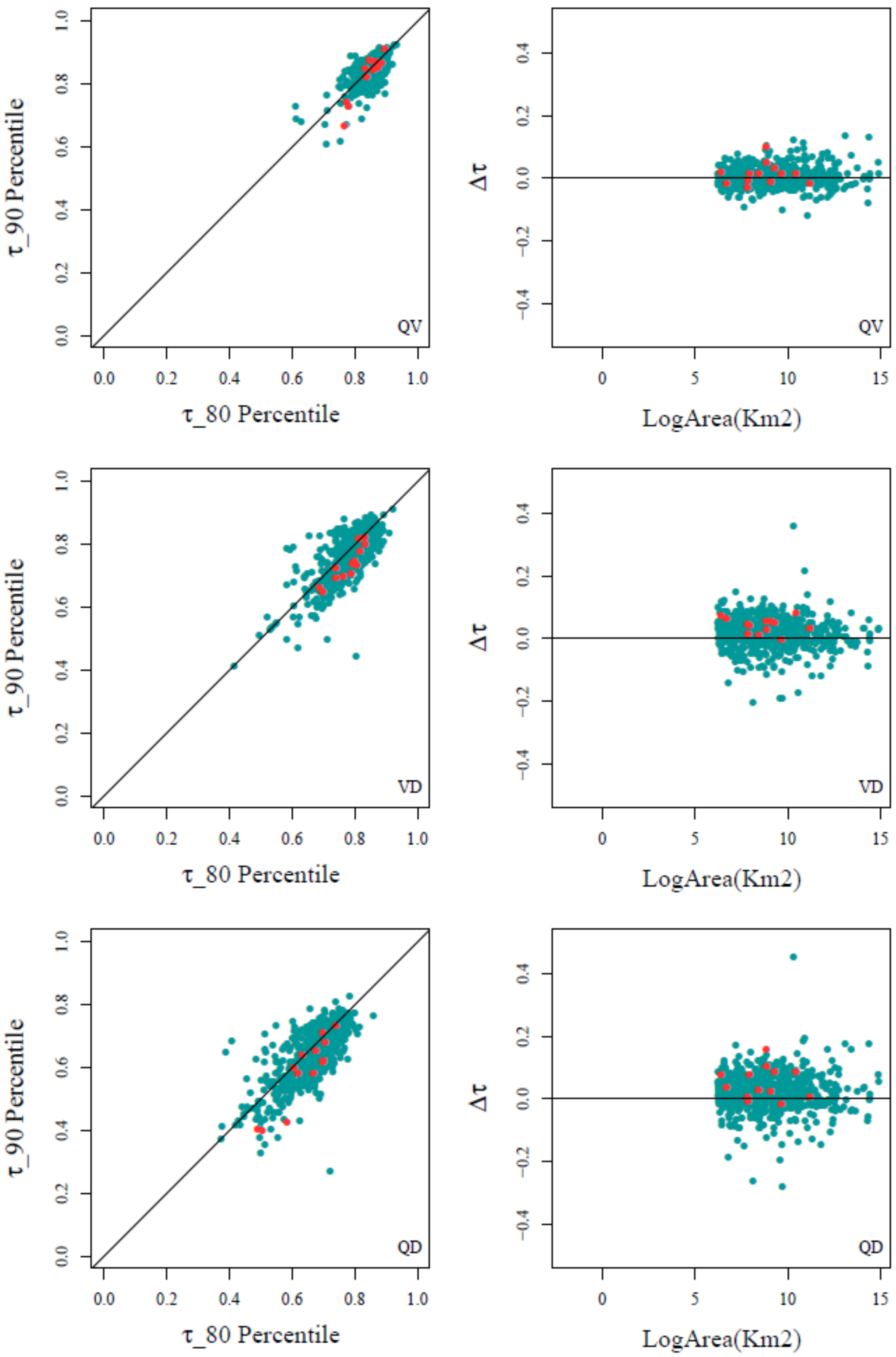

Figure S10. In the left panels, comparison of the Kendall's tau (for Q-V in the upper panels, V-D in the central panels, and Q-D in the lower panels) calculated considering two different thresholds to select flood events: 80th percentile and 90th percentile. In the right panels, the behavior of the difference of the two Kendall's taus, $\Delta\tau$, against the catchment area. Green points represent the worldwide dataset, while red points the U.S. subdataset considered in the analysis.

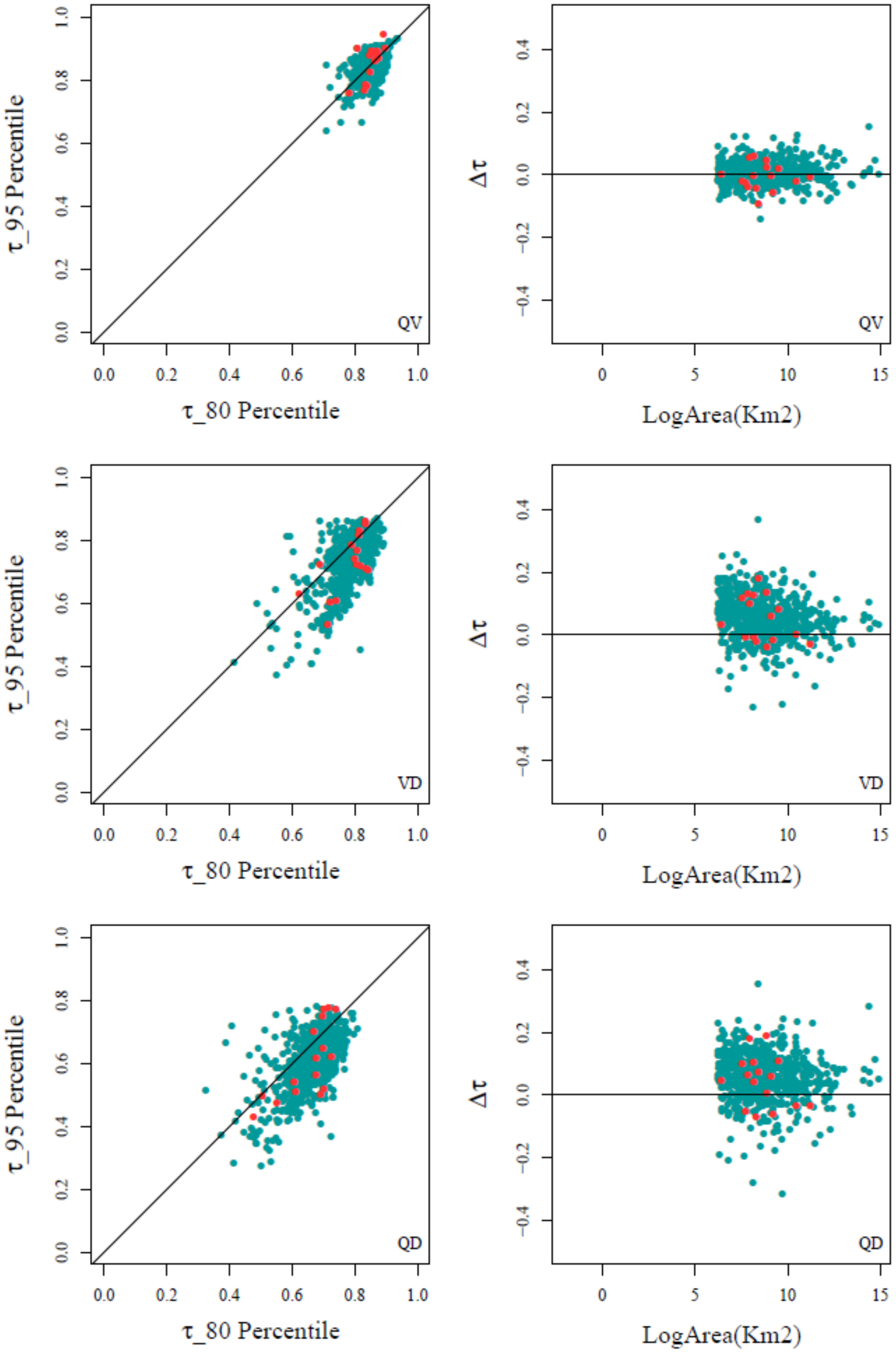

Figure S11. In the left panels, comparison of the Kendall's tau (for Q-V in the upper panels, V-D in the central panels, and Q-D in the lower panels) calculated considering two different thresholds to select flood events: 80th percentile and 95th percentile. In the right panels, the behavior of the difference of the two Kendall's taus, $\Delta\tau$, against the catchment area. Green points represent the worldwide dataset, while red points the U.S. subdataset considered in the analysis.

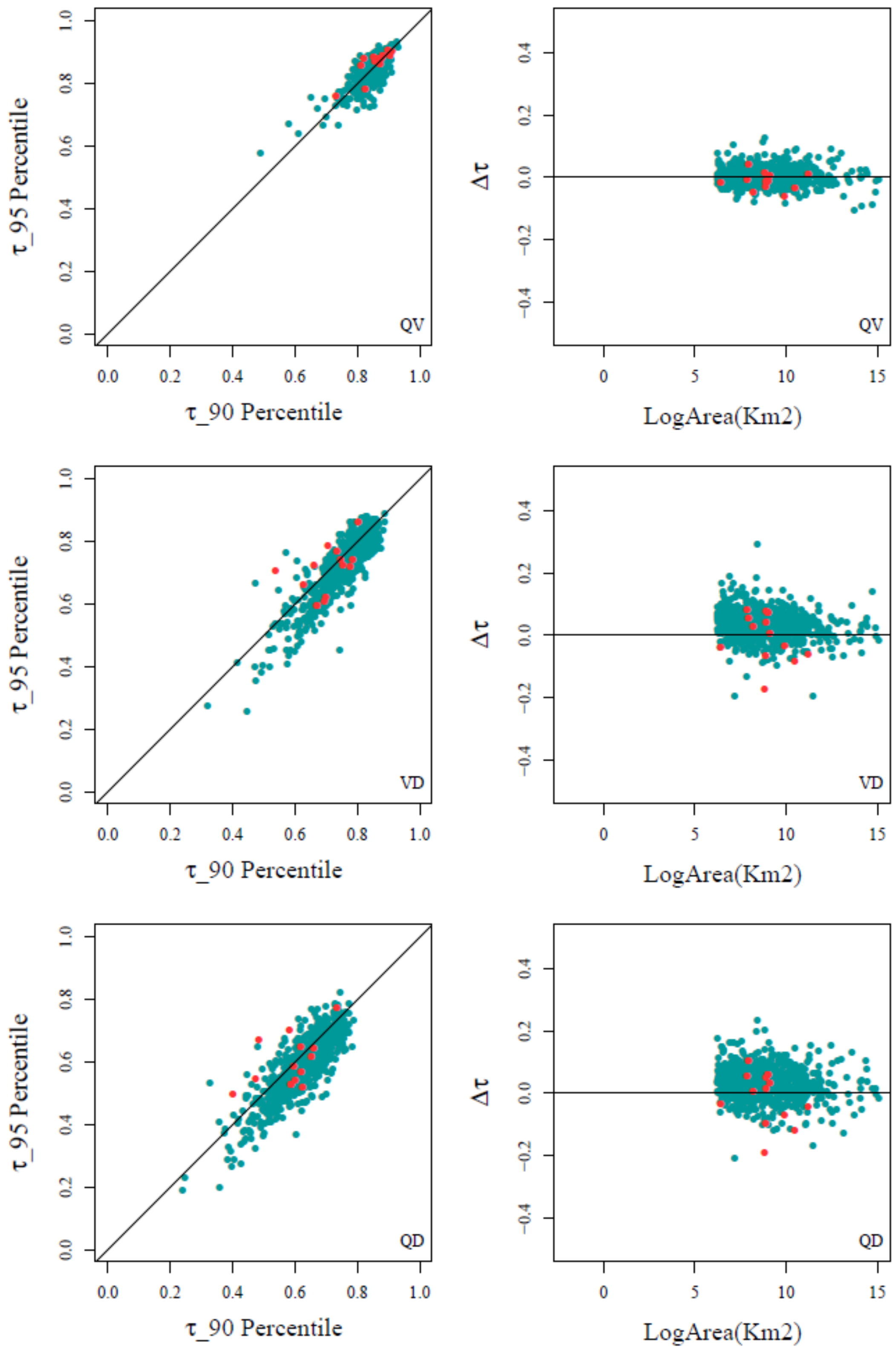

Figure S12. In the left panels, comparison of the Kendall's tau (for Q-V in the upper panels, V-D in the central panels, and Q-D in the lower panels) calculated considering two different thresholds to select flood events: 90th percentile and 95th percentile. In the right panels, the behavior of the difference of the two Kendall's taus, $\Delta\tau$, against the catchment area. Green points represent the worldwide dataset, while red points the U.S. subdataset considered in the analysis.

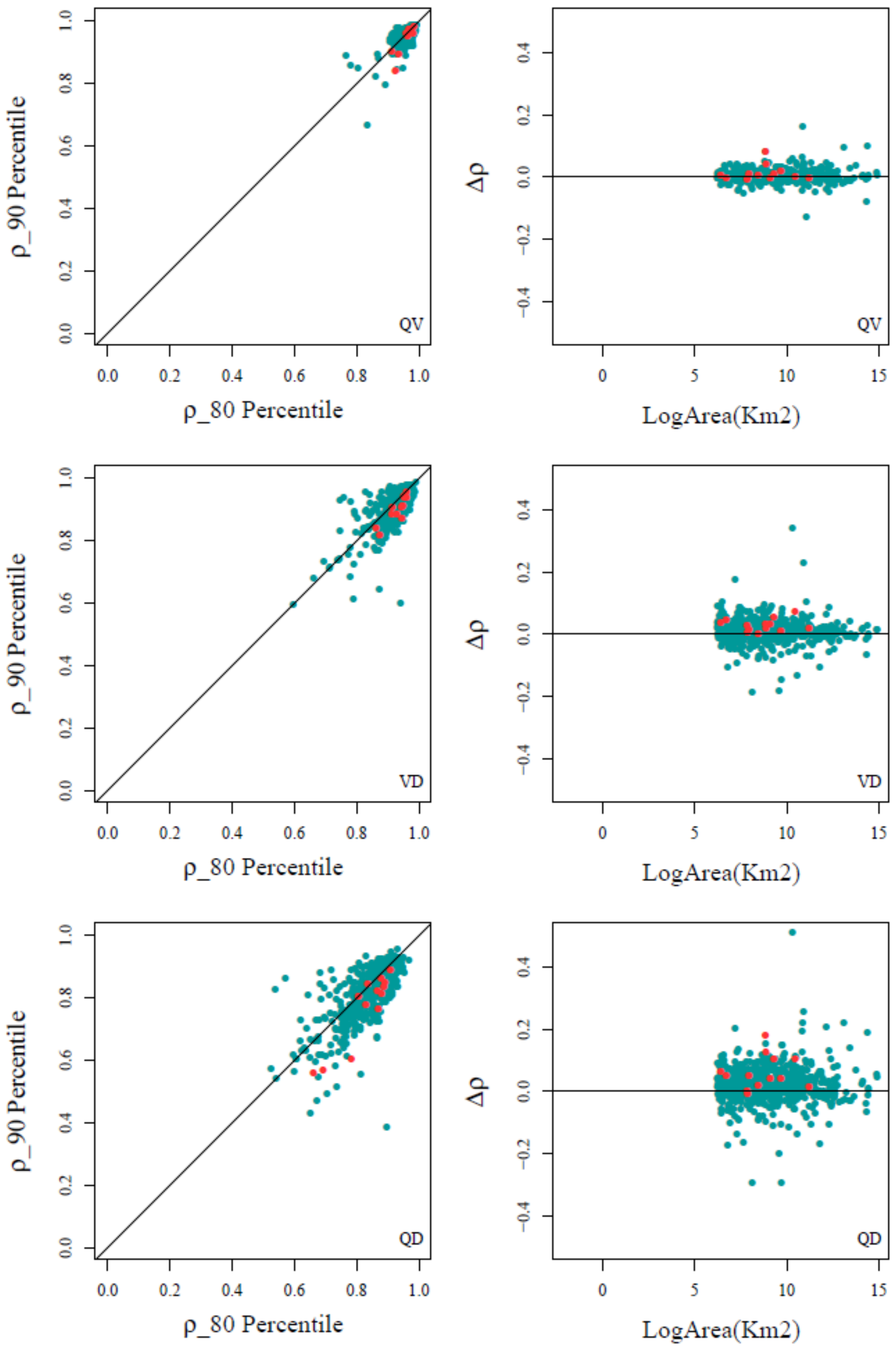

Figure S13. In the left panels, comparison of the Spearman's rho (for Q-V in the upper panels, V-D in the central panels, and Q-D in the lower panels) calculated considering two different thresholds to select flood events: 80th percentile and 90th percentile. In the right panels, the behavior of the difference of the two Spearman's rho correlations, Δρ, against the catchment area. Green points represent the worldwide dataset, while red points the U.S. subdataset considered in the analysis.

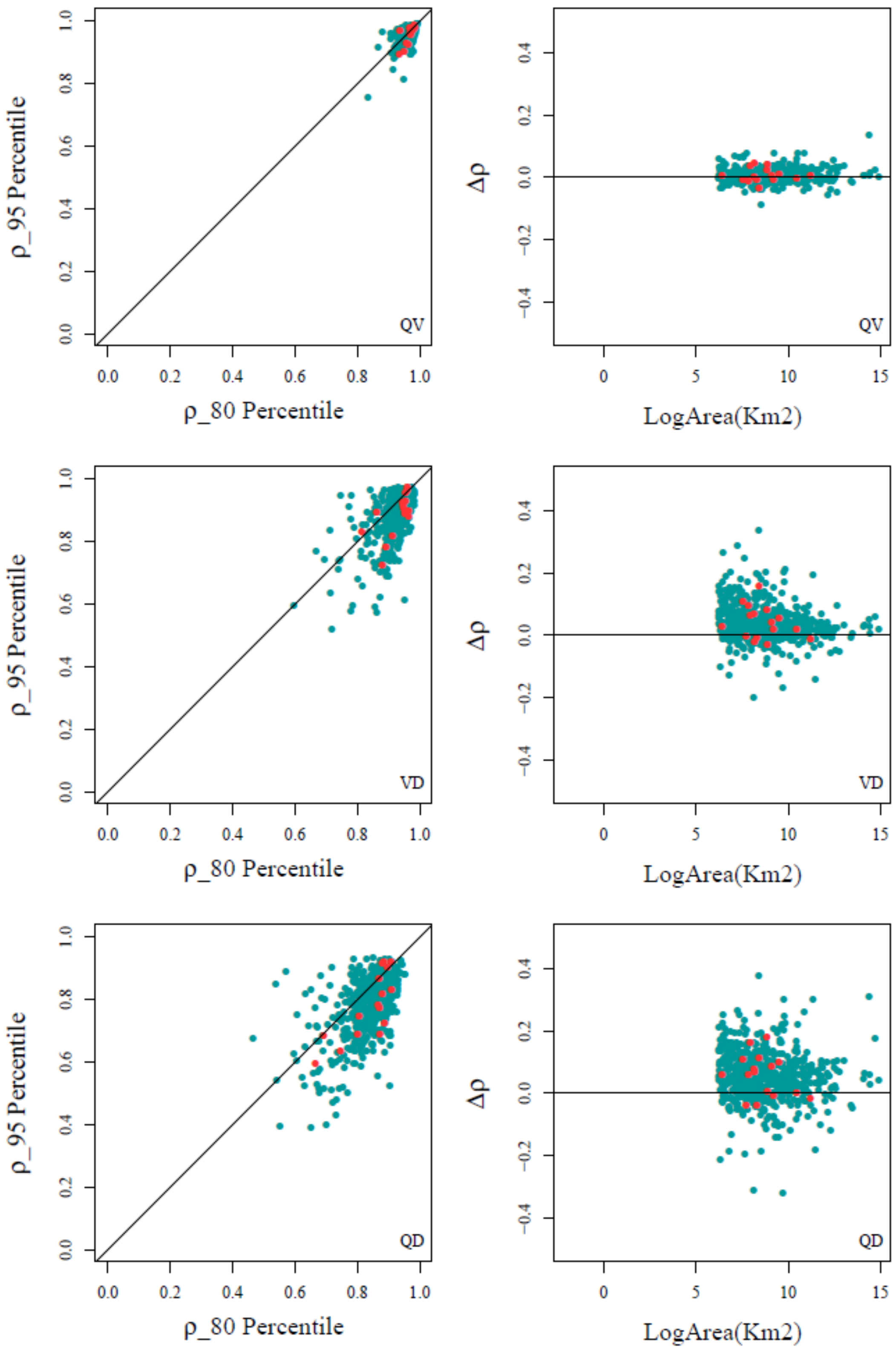

Figure S14. In the left panels, comparison of the Spearman's rho (for Q-V in the upper panels, V-D in the central panels, and Q-D in the lower panels) calculated considering two different thresholds to select flood events: 80th percentile and 95th percentile. In the right panels, the behavior of the difference of the two Spearman's rho correlations, Δρ, against the catchment area. Green points represent the worldwide dataset, while red points the U.S. subdataset considered in the analysis.

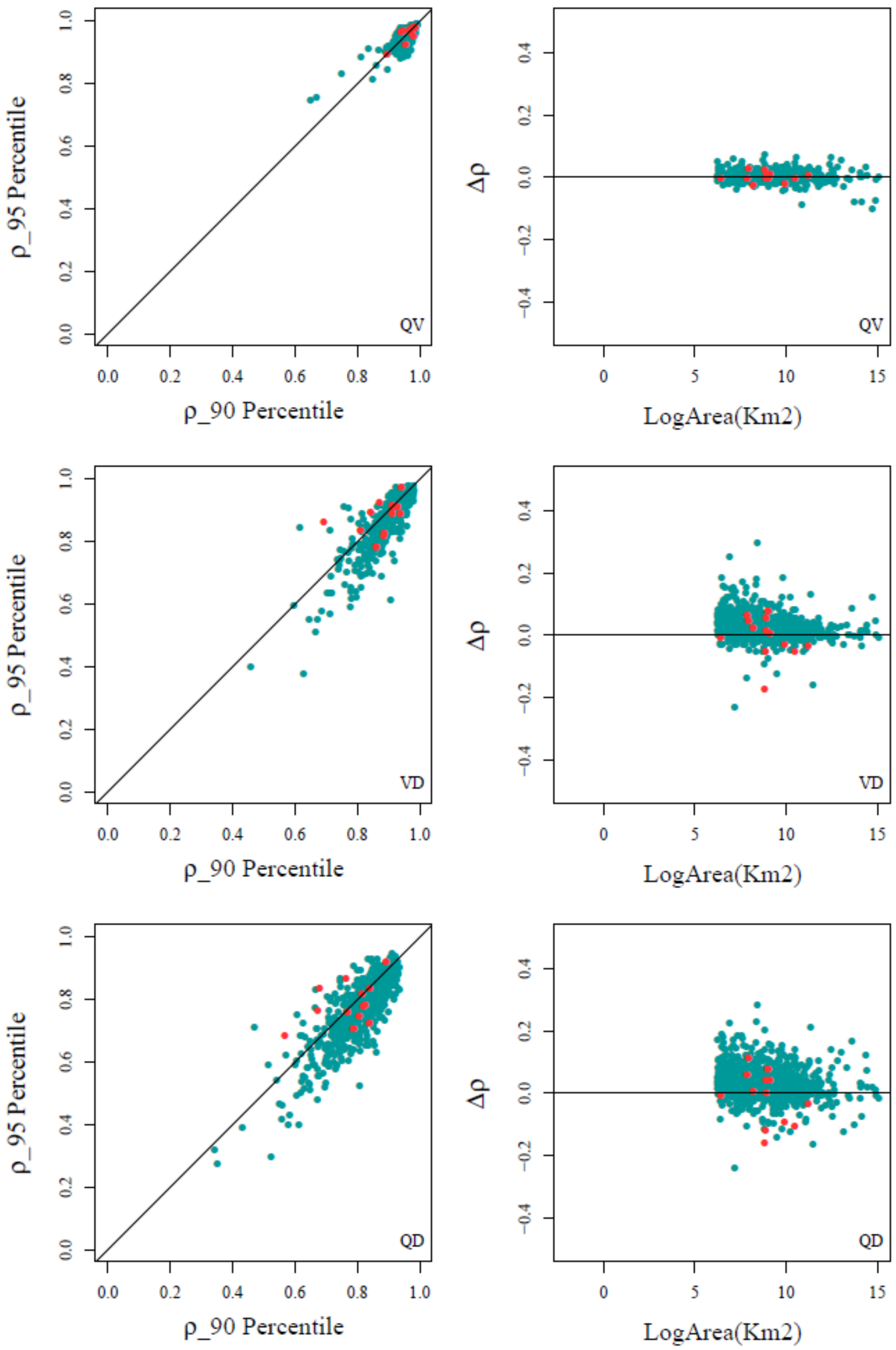

Figure S15. In the left panels, comparison of the Spearman's rho (for Q-V in the upper panels, V-D in the central panels, and Q-D in the lower panels) calculated considering two different thresholds to select flood events: 90th percentile and 95th percentile. In the right panels, the behavior of the difference of the two Spearman's rho correlations, Δρ, against the catchment area. Green points represent the worldwide dataset, while red points the U.S. subdataset considered in the analysis.

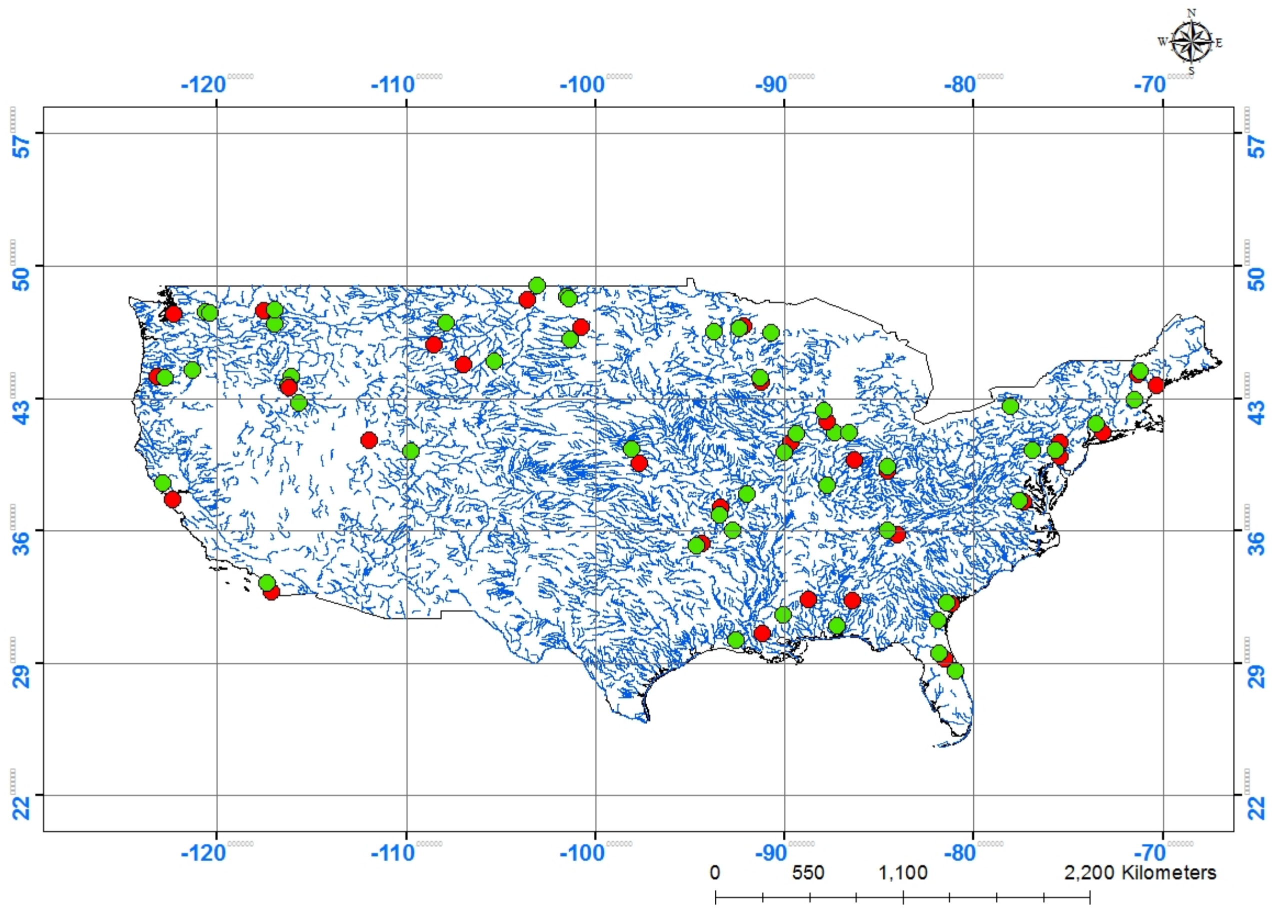

Figure S16. Location of rain gauges (red points) and discharge stations (green points). Each green point represents the outlet of a basin, where the closest red point is the representative rain gauge.

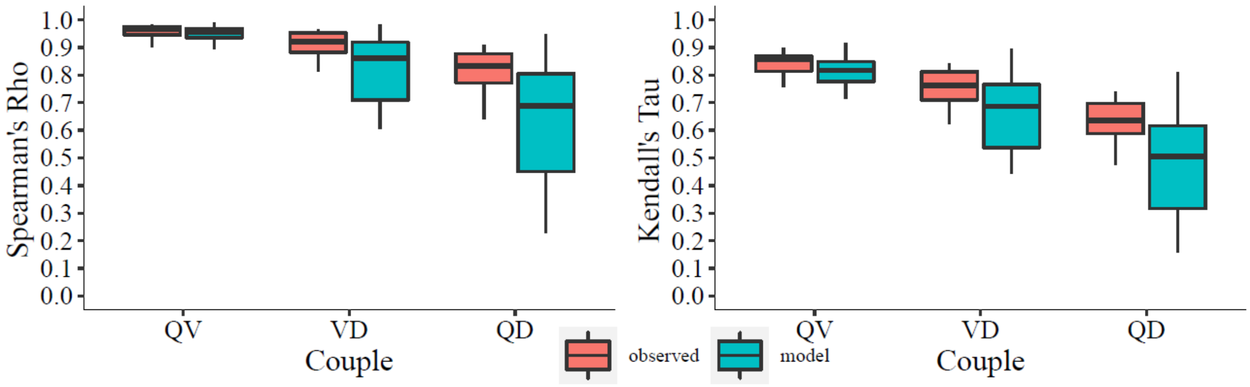

Figure S17. Comparison between observed (using 80th percentile of daily discharge as threshold) and simulated pairwise dependencies in terms of boxplots, for U.S. subdataset. Spearman's rho is in the left panel, and the Kendall's tau in the right panel.

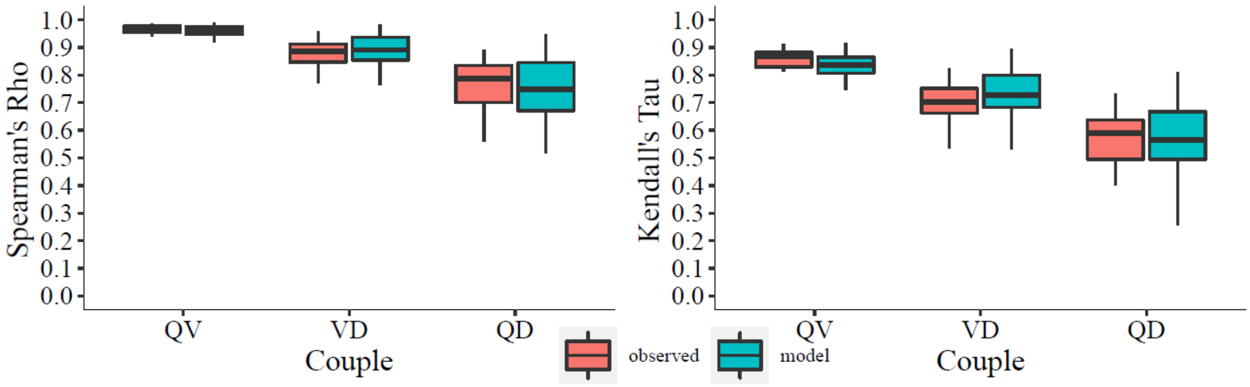

Figure S18. Comparison between observed (using 95th percentile of daily discharge as threshold) and simulated pairwise dependencies in terms of boxplots, for U.S. subdataset. Spearman's rho is in the left panel, and the Kendall's tau in the right panel.
